# Supplementary material for: Prediction of the 3D cancer genome from whole-genome sequencing using InfoHiC
Source: Mol Syst Biol. 2024 Sep 25;20(11):1156–72. doi: 10.1038/s44320-024-00065-2 (PMC11535030; doi:10.1038/s44320-024-00065-2)
Supplement: Supplementary file 1 — Appendix [file 44320_2024_65_MOESM1_ESM.pdf]

Appendix for prediction of the 3D cancer genome from whole-genome sequencing using InfoHiC

Yeonghun Lee[1], Sung-Hye Park[2,3,4] & Hyunju Lee[1,5,*]

[1] *School of Electrical Engineering and Computer Science, Gwangju Institute of Science and Technology, Gwangju 61005, South Korea.*

[2] *Department of Pathology, Seoul National University Hospital, Seoul National University College of Medicine, 103 Daehak-ro, Jongno-gu, Seoul, 03080, South Korea*

[3] *Institute of Neuroscience, Seoul National University Hospital, Seoul, Republic of Korea*

[4] *Department of Pathology, Seoul National University College of Medicine, Seoul, 03080, Republic of Korea*

[5] *AI Graduate School, Gwangju Institute of Science and Technology, Gwangju 61005, South Korea.*

**Corresponding author*

Email: hyunjulee@gist.ac.kr for Hyunju Lee

Table of Contents

Appendix Figures

Appendix Figure S1. Comparison between InfoHiC and deepC on reference windows 4

Appendix Figure S2. Comparison between InfoHiC and deepC-SV on SV windows 5

Appendix Figure S3. Performance comparison between InfoHiC and deepC for the training usage of HMEC and MCF10A 6

Appendix Figure S4. The Hi-C prediction result of InfoHiC at a 10-kb resolution in the K562 cell line 7

Appendix Figure S5. InfoHiC validation scheme for examples of neo-TADs and SE hijacking events 8

Appendix Figure S6. Gene expression according to integer CNs in breast cancer cell lines 9

Appendix Figure S7. The SE hijacking event of the *IDO1* gene in the HCC1954 cell line 10

Appendix Figure S8. Comparison of gene-SE pairs in predicted neo-TADs between deepC-SV and InfoHiC 11

Appendix Figure S9. InfoHiC prediction of contig Hi-C matrices of the *LASP1* region in BRCA patients 13

Appendix Figure S10. 1D genome and 3D genome analysis of the PD2105 patient 14

Appendix Figure S11. 1D genome and 3D genome analysis of the PD2107 patient 15

Appendix Figure S12. 1D genome and 3D genome analysis of the PD2109 patient 16

Appendix Figure S13. 1D genome and 3D genome analysis of the PD2110 patient 17

Appendix Figure S14. SE hijacking events of *GFI1* gene families in patients with medulloblastoma 18

Appendix Figure S15. Comparison of neo-loop-involved genes found by NeoLoopFinder and InfoHiC 19

Appendix Figure S16. Prediction results using the fine-tuned models on medulloblastoma driver

genes 21

Appendix Tables

Appendix Table S1. InfoHiC performance for reference windows of the test set 1 22

Appendix Table S2. InfoHiC performance for reference windows and SV windows of the test set 2 23

Appendix Table S3. Cancer-related genes of Neo-TADs found in breast cancer cell lines 24

Appendix Table S4. Gene expression in neo-TADs and neo-loops in breast cancer cell lines 25

Appendix Table S5. DeepC-SV specific cancer related genes involved with SE hijacking events and their Hi-C intensities 26

Appendix Table S6. InfoHiC-specific SE hijacking events of cancer related genes and their Hi-C intensities 27

Appendix Table S7. Gene expression in neo-TADs and neo-loops in BRCA datasets 28

Appendix Table S8. Recurrent neo-TAD and enhancer hijacking genes with overexpression 29

Appendix Table S9. Exonic SNVs and indels found in patients with medulloblastoma 30

Appendix Table S10. CNAs found in patients with medulloblastoma 35

Appendix Table S11. SEdb Samples used for SE and TE annotation 40

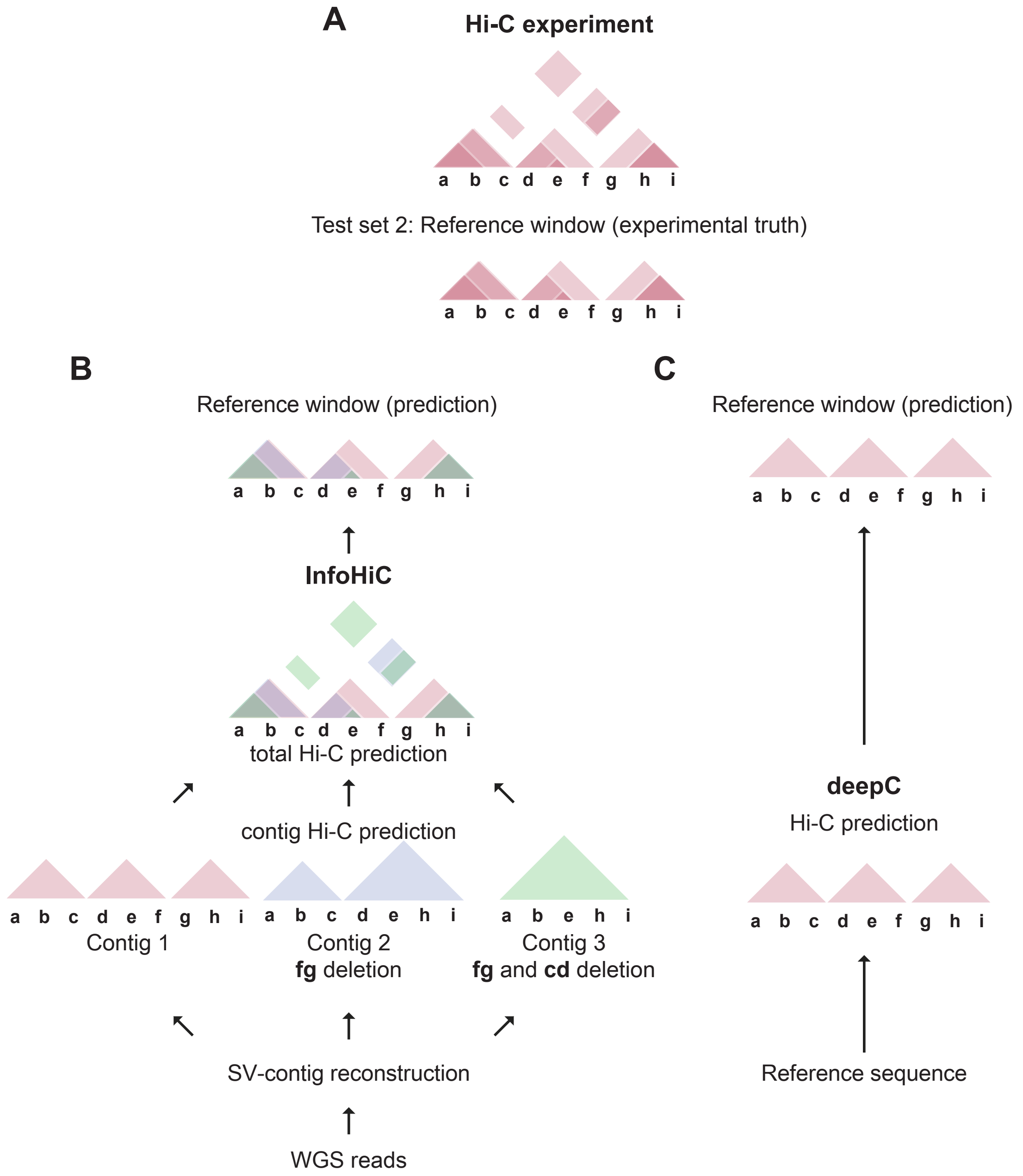

Appendix Figure S1. Comparison between InfoHiC and deepC on reference windows. InfoHiC and deepC use different strategies for Hi-C prediction on reference windows. (**A**) The Hi-C experiment truth is shown on the reference window of the test set 2. (**B**) InfoHiC utilizes WGS and reconstruct SV contigs, resulting in three SV contigs (Contig 1 to 3). Contig 1 is the same with the reference (red), Contig 2 has the fg deletion (blue), and Contig 3 has the fg and cd deletion together (green). (**C**) In contrast, deepC uses the reference sequence for the reference window prediction. No genomic variation is encoded in deepC. InfoHiC validates Hi-C prediction results on the total Hi-C coordinate, where Hi-C intensities from SV contigs are summed together. Note that even in the reference window, CNAs and SVs can change Hi-C intensities.

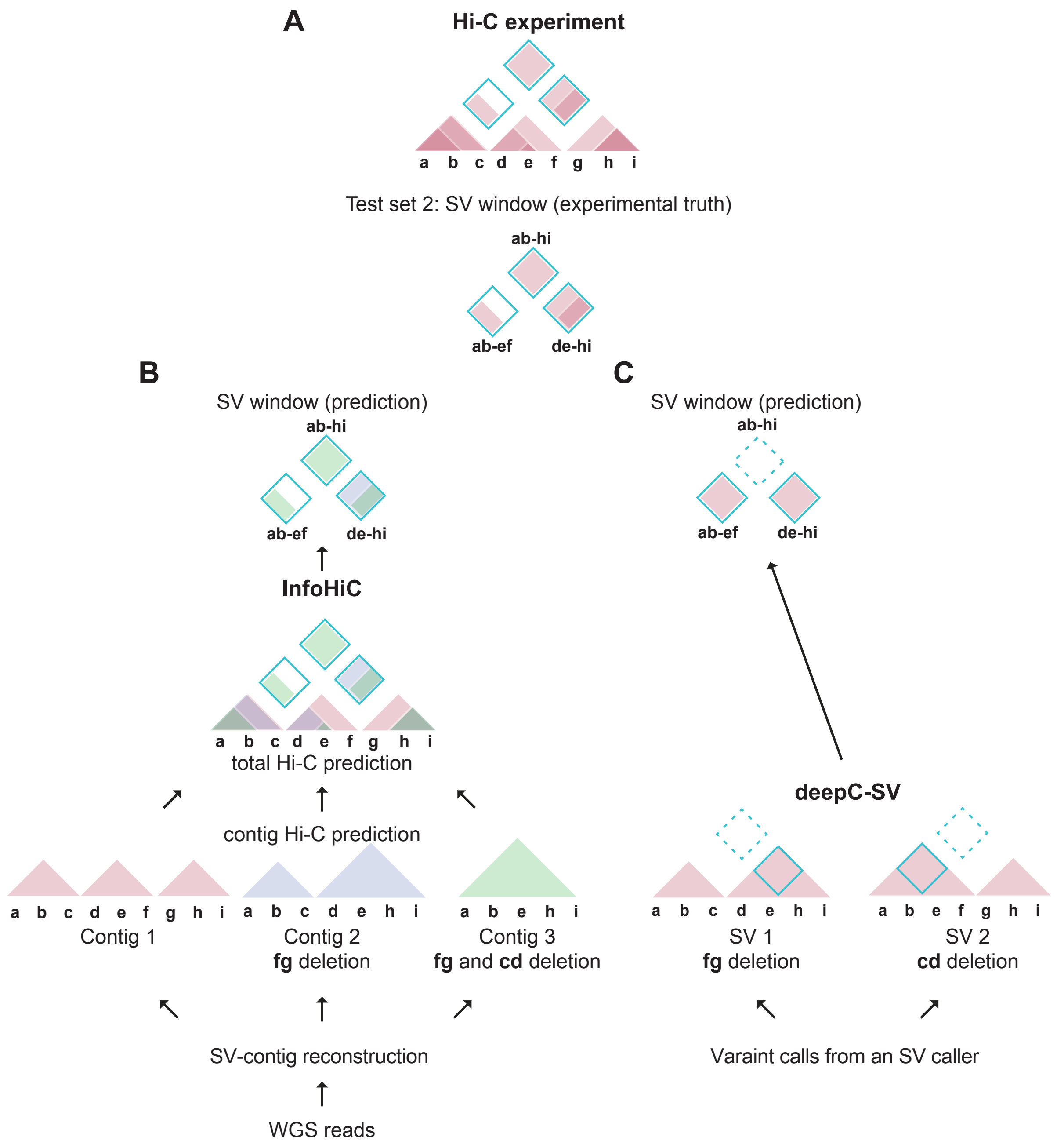

Appendix Figure S2. Comparison between InfoHiC and deepC-SV on SV windows. InfoHiC and deepC-SV use different strategies for Hi-C prediction on SV windows. (**A**) The Hi-C experiment truth is shown on the SV window of the test set 2. (**B**) InfoHiC utilizes WGS and reconstruct SV contigs, resulting in three SV contigs (Contig 1 to 3). Contig 1 is the same with the reference (red), Contig 2 has the fg deletion (blue), and Contig 3 has the fg and cd deletion together (green). (**C**) In contrast, deepC-SV uses SV calls from an SV caller, and performs prediction on each SV call (the fg deletion and cd deletion, respectively). InfoHiC validates Hi-C prediction results on the total Hi-C coordinate, where Hi-C intensities from SV contigs are summed together. Three SV windows can be validated (ab-ef, de-hi, and ab-hi windows). Note that Hi-C contacts (the e-h contact and e-i contact) from Contig 2 (the de-hi TAD) and Contig 3 (the ab-e-hi TAD) are overlapped in the de-hi SV window. The ab-hi window is not verifiable in the deepC-SV.

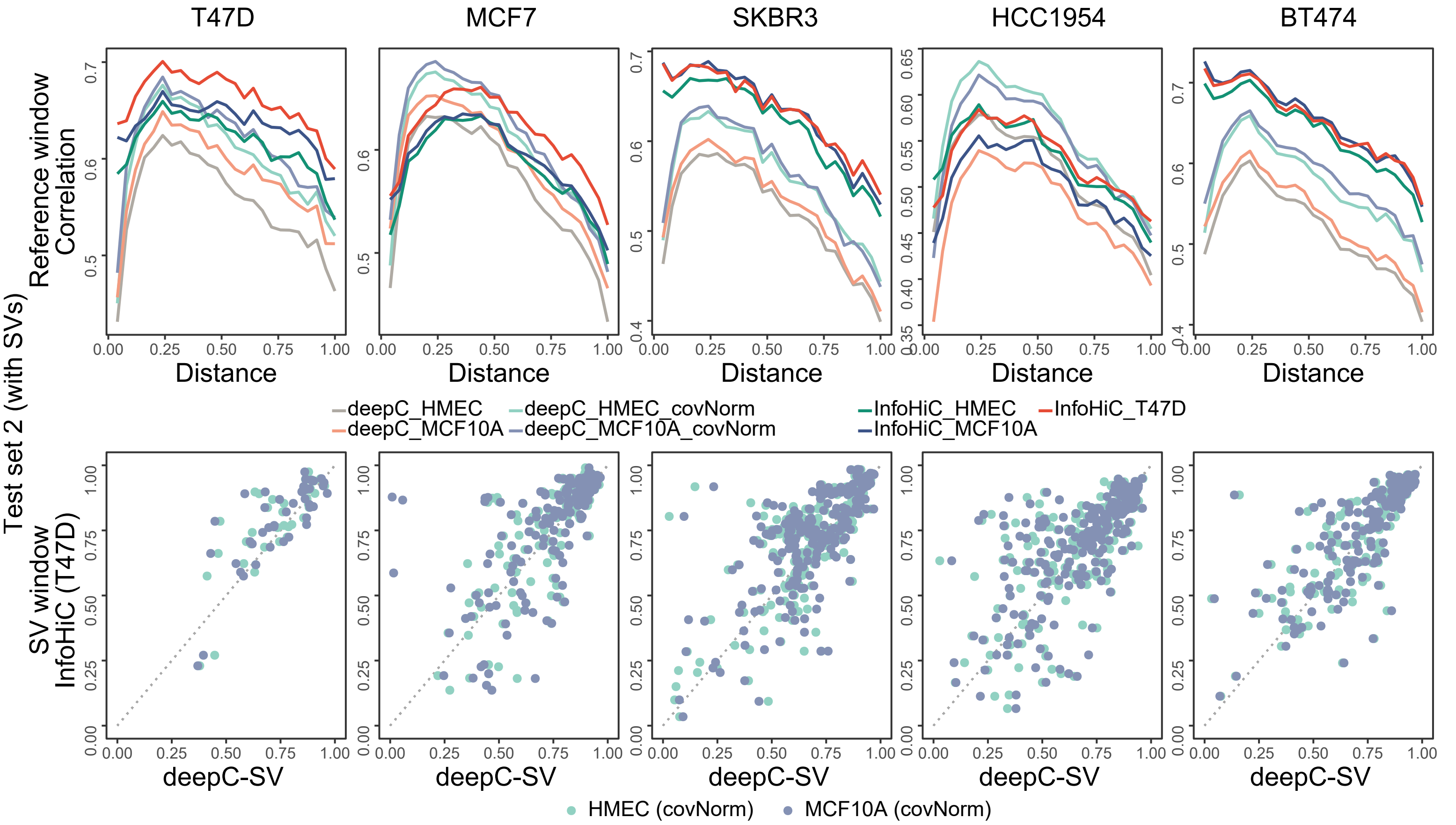

Appendix Figure S3. Performance comparison between InfoHiC and deepC for the training usage of HMEC and MCF10A. InfoHiC and deepC are compared for the training usage of the T47D, HMEC, and MCF10A cell line using the test set 2. The implicit normalization (covNorm) for deepC was applied to the HMEC and MCF10A Hi-C data. Distance-stratified correlations (line) for reference windows are shown (top) according to the mega-base scale distance range (0-1 Mb), and Pearson correlation (dot) per each SV window (bottom) is compared between the best models, deepC-SV trained by implicitly-normalized HMEC or MCF10A Hi-C data (bottom) and InfoHiC trained by the raw T47D Hi-C data (left).

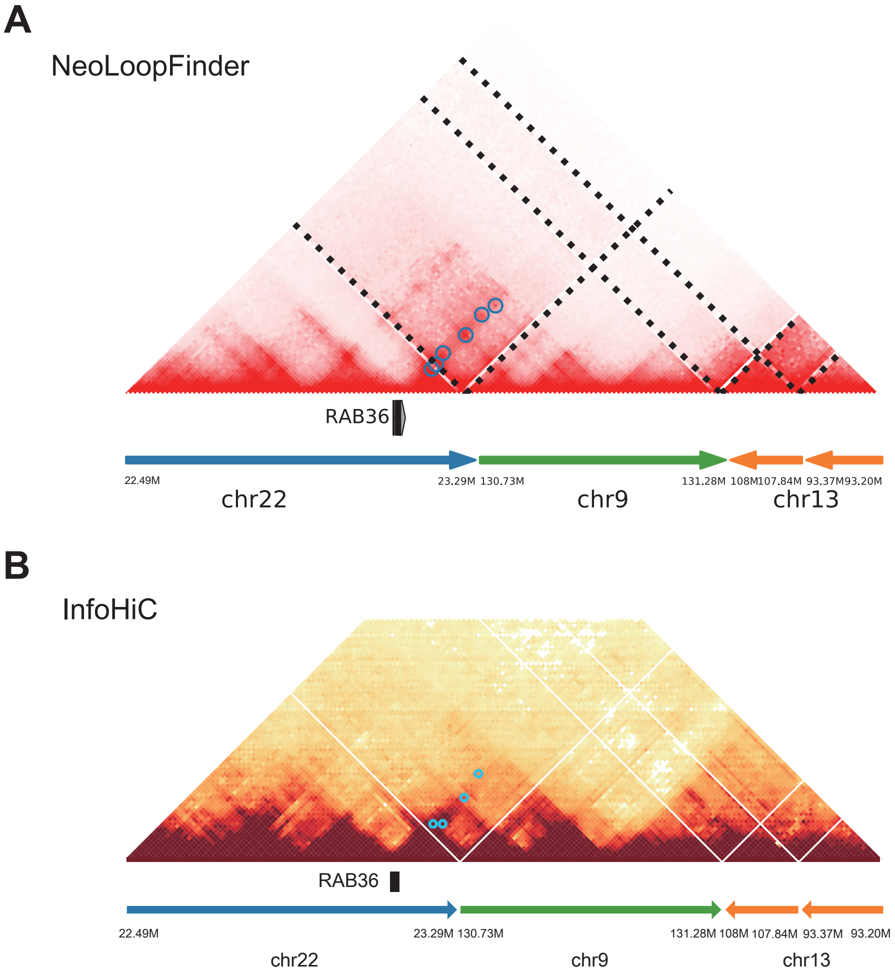

Appendix Figure S4. The Hi-C prediction result of InfoHiC at a 10-kb resolution in the K562 cell line. NeoLoopFinder and InfoHiC results on the inter-chromosomal SV contig (chromosomes 9, 13, and 22) in the K562 cell line. (**A**) NeoLoopFinder (top) manipulated the observed Hi-C experiment to reconstruct the contig Hi-C matrix, and annotated neo-loops (blue) around the *RAB36* gene. The neoLoopFinder was based on the hg38 reference. (**B**) To compare with the InfoHiC result from the hg19 reference, we performed Hi-C prediction on the SV contig lift-overed into the hg38 reference (bottom). InfoHiC annotated neo-loops (cyan) around the *RAB36* gene.

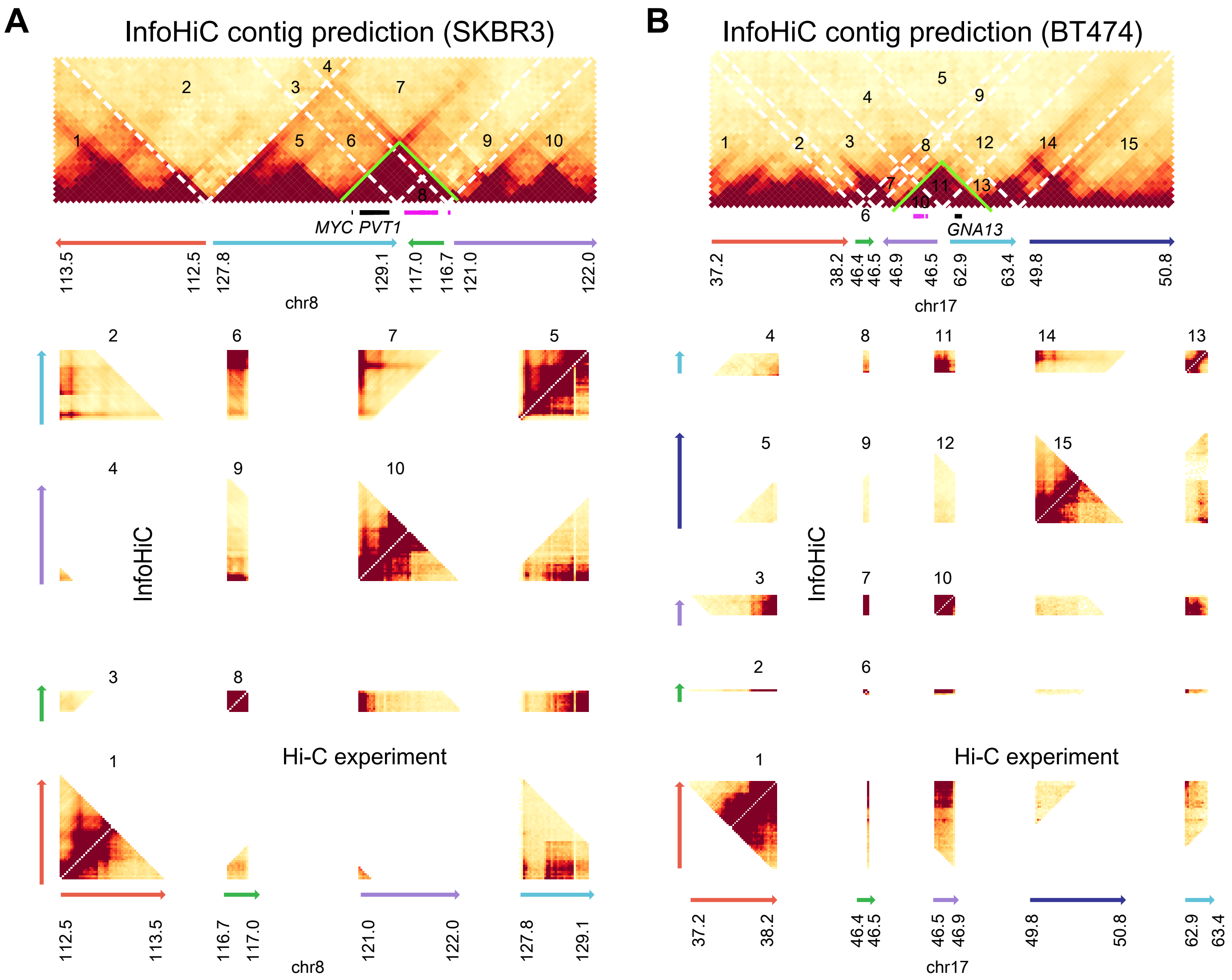

Appendix Figure S5. InfoHiC validation scheme for examples of neo-TADs and SE hijacking events. (**A**) The InfoHiC result of the neo-TAD prediction of the *MYC* and *PVT1* genes found in the SKBR3 cell line. (**B**) The InfoHiC result of the neo-TAD prediction of the *GNAS* gene found in the BT474 cell line. The contig Hi-C matrix has SV-derived contacts and each SV window is numbered. Dotted white lines represent SV breakpoints. Reference coordinates are shown below the contig Hi-C matrix at the megabase scale with the gene (black) and SE (crimpson) annotation. The total Hi-C matrix on the reference coordinate is shown at the bottom. The upper diagonal matrix is InfoHiC prediction and the lower diagonal matrix is the Hi-C experiment of the corresponding cell ilne. SV windows numbered from the contig Hi-C matrix are compared with Hi-C observation in the reference coordinate.

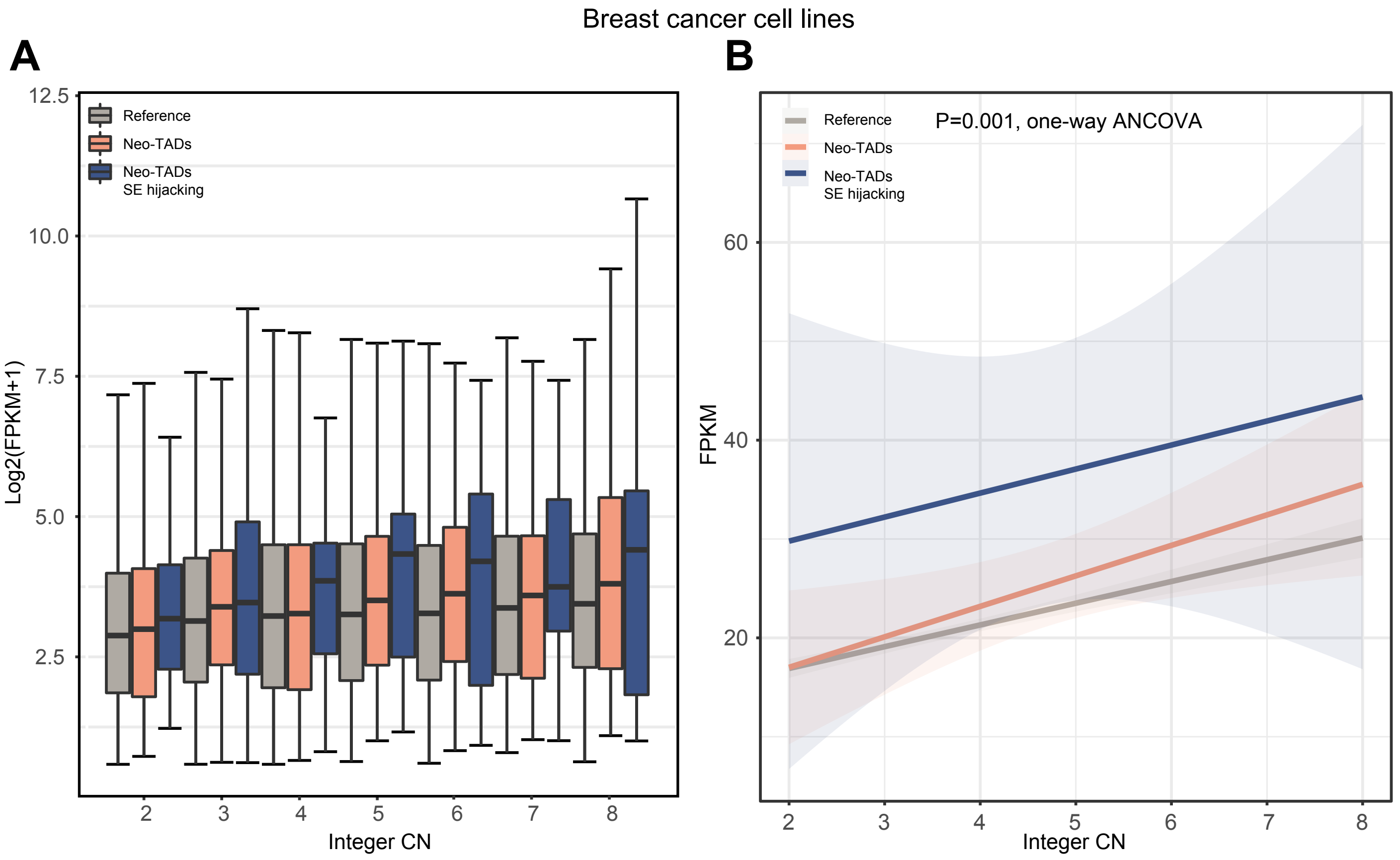

Appendix Figure S6. Gene expression according to integer CNs in breast cancer cell lines. (**A**) Boxplots of RNA-seq Log2(FPKM+1) values depending on neo-TAD classes are shown according to integer CNs (left). The boxplot centre lines are medians, box limits are upper and lower quantiles, and wihskers are 1.5x interquantile ranges. (**B**) The regression line between integer CNs and FPKM values is shown with the confidence interval for each neo-TAD class, and the P value was calculated by the one-way ANCOVA.

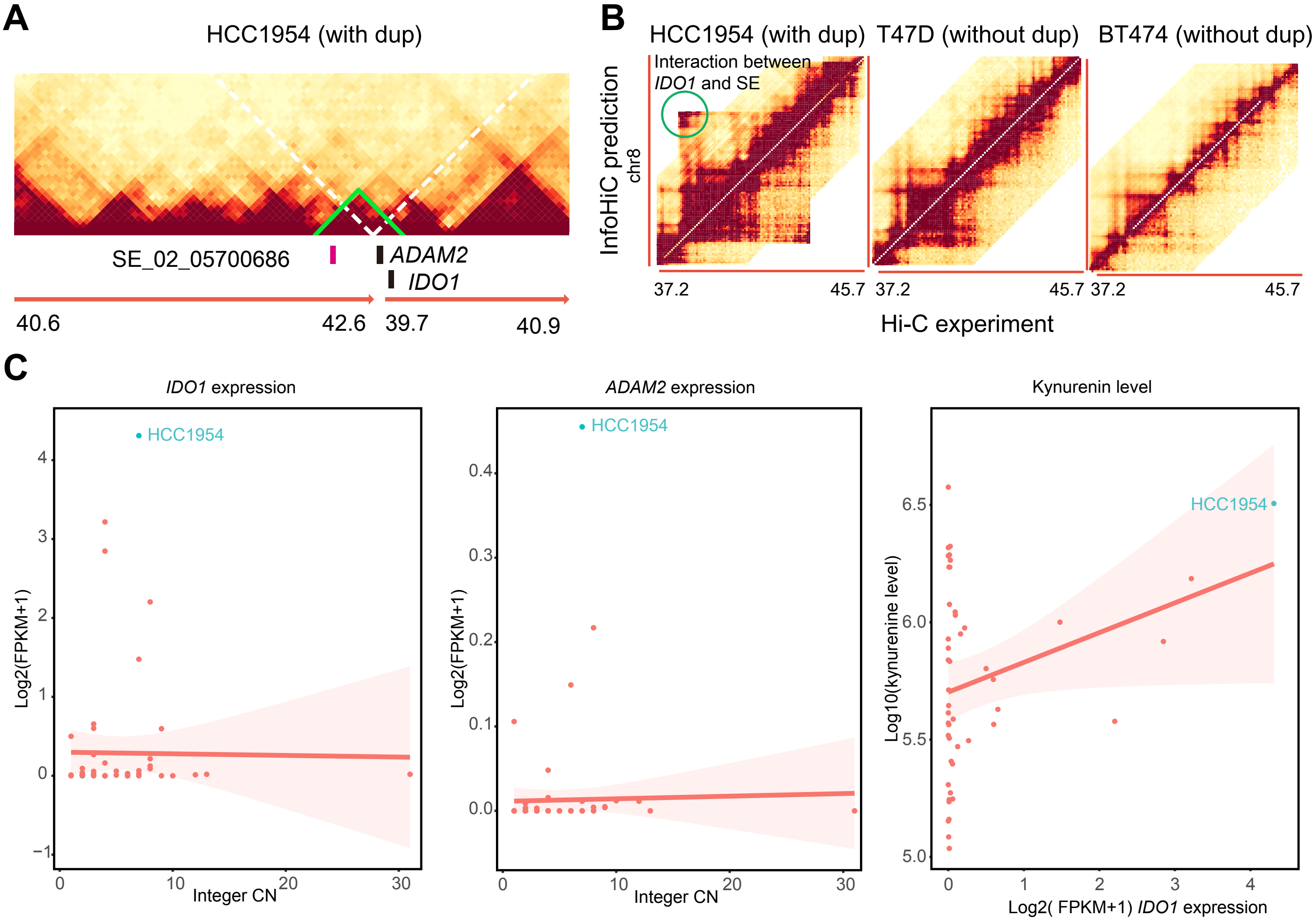

Appendix Figure S7. The SE hijacking event of the *IDO1* gene in the HCC1954 cell line.. (**A**) The tandem duplication forms a neo-TAD (green) and results in the SE hijacking (crimpson) of the *IDO1* and *ADAM2* gene. (**B**) In the reference coordinate, the InfoHiC prediction of the SE hijacking event was validated by the HCC1954 Hi-C experiment, and it was a novel interaction that is not observed in other cell lines without the duplication (T47D and BT474). (**C**) Log2(FPKM+1) values of the *IDO1* and *ADAM2* gene are plotted versus integer CNs, and log10 values of the kynerenine level are plotted versus Log2(FPKM+1) value of the *IDO1* gene. Regression lines are shown with confidence intervals (red), and values from the HCC1954 cell line are shown in cyan.

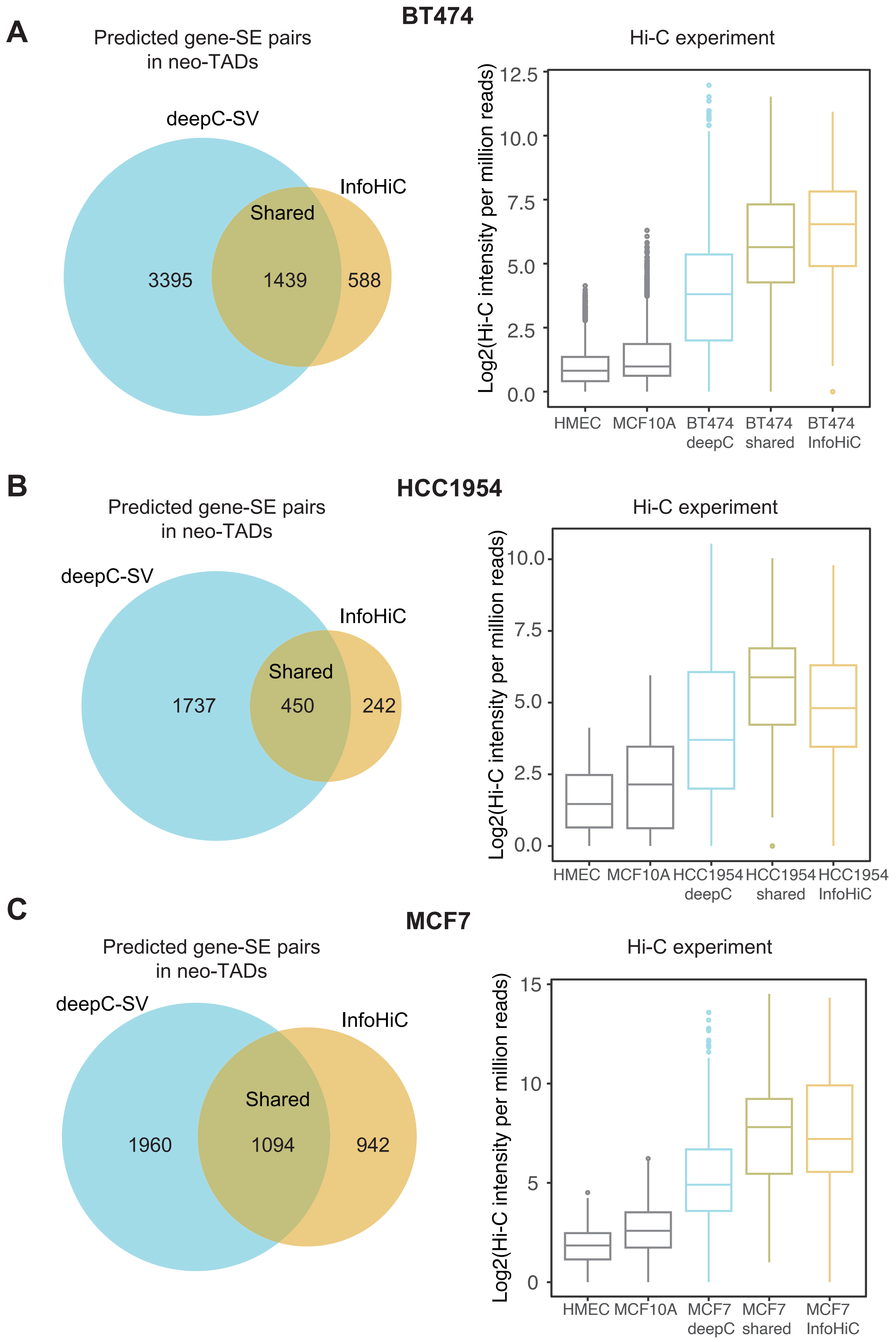
A
BT474
Predicted gene-SE pairs
in neo-TADs
deepC-SV
InfoHiC
Shared
3395
1439
588
Hi-C experiment
Log2(Hi-C intensity per million reads)
12.5
10.0
7.5
5.0
2.5
0.0
HMEC
MCF10A
BT474 deepC
BT474 shared
BT474 InfoHiC
B
HCC1954
Predicted gene-SE pairs
in neo-TADs
deepC-SV
InfoHiC
Shared
1737
450
242
Hi-C experiment
Log2(Hi-C intensity per million reads)
10.0
7.5
5.0
2.5
0.0
HMEC
MCF10A
HCC1954 deepC
HCC1954 shared
HCC1954 InfoHiC
C
MCF7
Predicted gene-SE pairs
in neo-TADs
deepC-SV
InfoHiC
Shared
1960
1094
942
Hi-C experiment
Log2(Hi-C intensity per million reads)
15
10
5
0
HMEC
MCF10A
MCF7 deepC
MCF7 shared
MCF7 InfoHiC

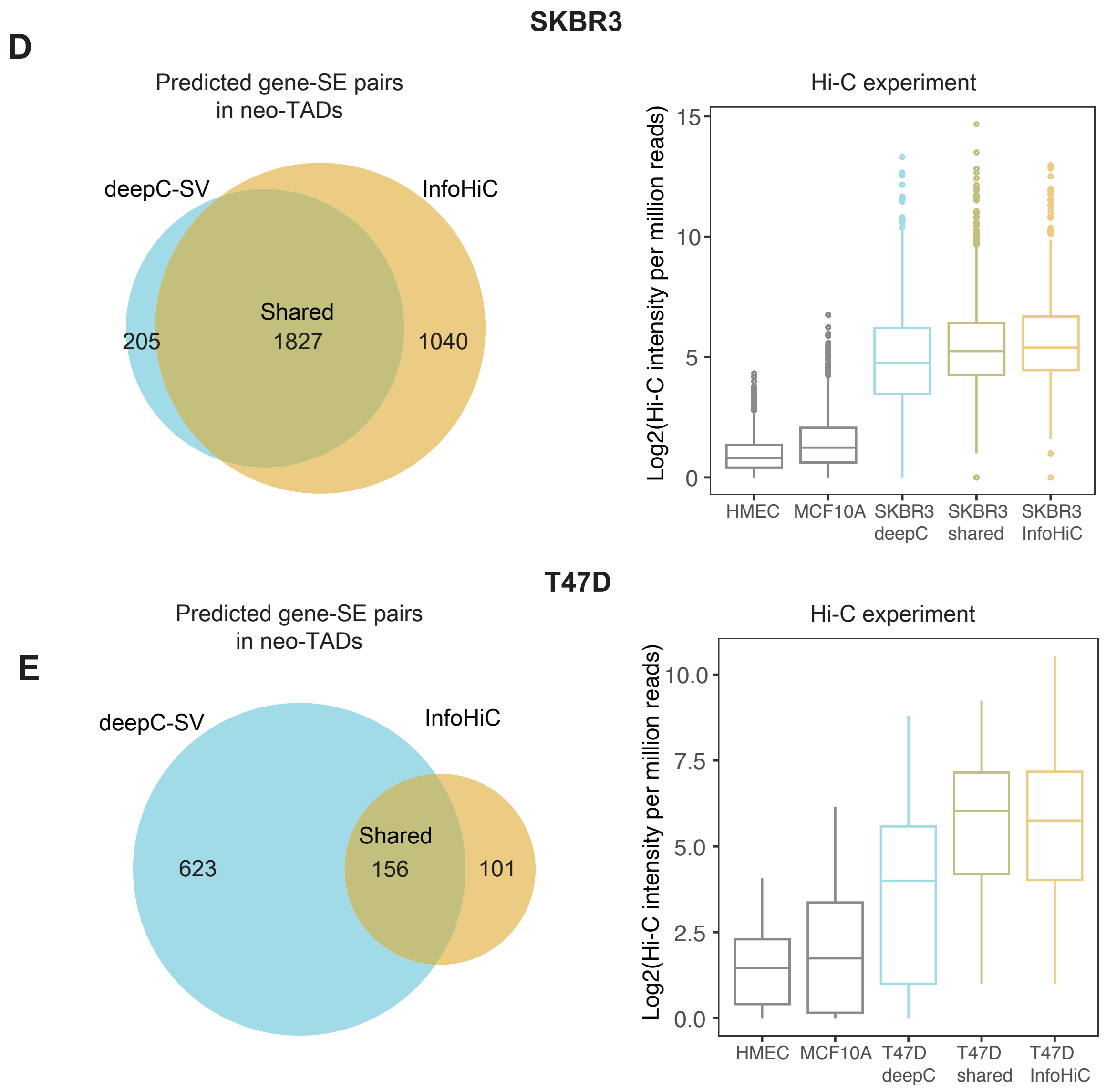

Appendix Figure S8. Comparison of gene-SE pairs in predicted neo-TADs between deepC-SV and InfoHiC.. A Venn diagram (left) of predicted gene-SE pairs and Hi-C intensities in the Hi-C experiment (right) for each cancer cell line. Cancer cell lines include (**A**) BT474, (**B**) HCC1954, (**C**) MCF7, (**D**) SKBR3, and (**E**) T47D. The Venn diagram (left) shows portions of deepC-SV specific (cyan), InfoHiC specific (orange), and shared events between them (brown). The box plot (right) shows Hi-C intensities observed in the cancer Hi-C experiment. Hi-C intensities of predicted gene-SE pairs (either by deepC-SV or InfoHiC, or shared) in HMEC and MCF10A experiments were measured as controls. The intensities were normalized as read counts per million Hi-C reads. Box plot colors represent events predicted by deepC-SV (blue), InfoHiC (orange), and shared events between them (brown). Boxplot center lines are medians, box limits are upper and lower quartiles, and dots are outliers.

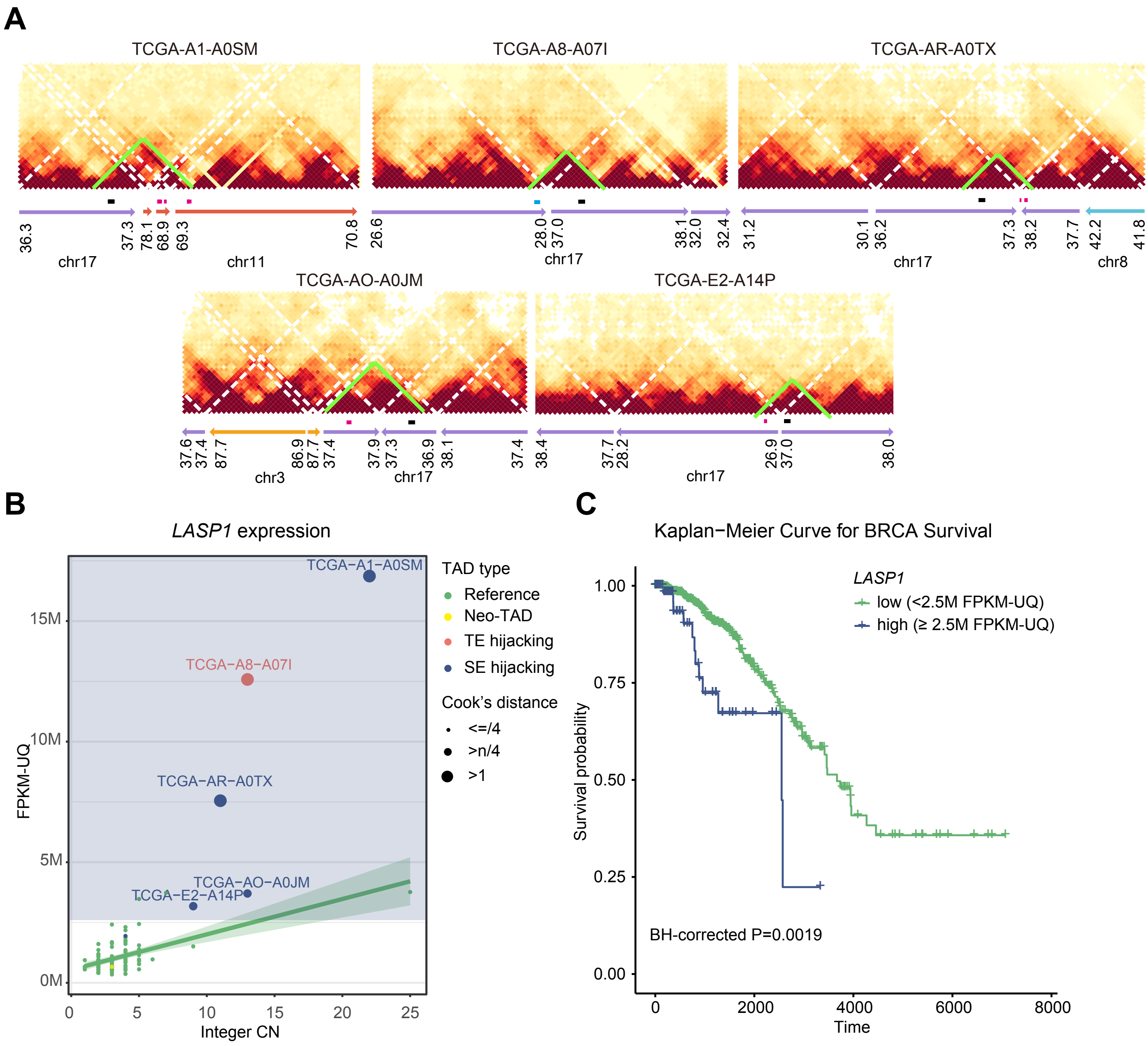

Appendix Figure S9. InfoHiC prediction of contig Hi-C matrices of the *LASP1* region in BRCA patients.. (**A**) Neo-TADs are shown in green with hijacked SEs (crimson), hijacked TEs (blue), and the *LASP1* gene (black). (**B**) Gene expression of *LASP1* of BRCA patients (dot) with WGS data (n=90). A linear regression line between FPKM-UQ values and integer CNs is shown with a confidence interval range (green). Neo-TADs are shown in different colors according to TAD types and in a different circle size according to the Cook's distance. The cutoff region of the FPKM-UQ value for overexpression is colored in the backgound (blue). (**C**) Kaplan-Meier curve for BRCA survival with RNA-seq data (n=1098). The P value was calculated by the log-rank test, and adjusted by the BH procedure across neo-TAD overexpression genes.

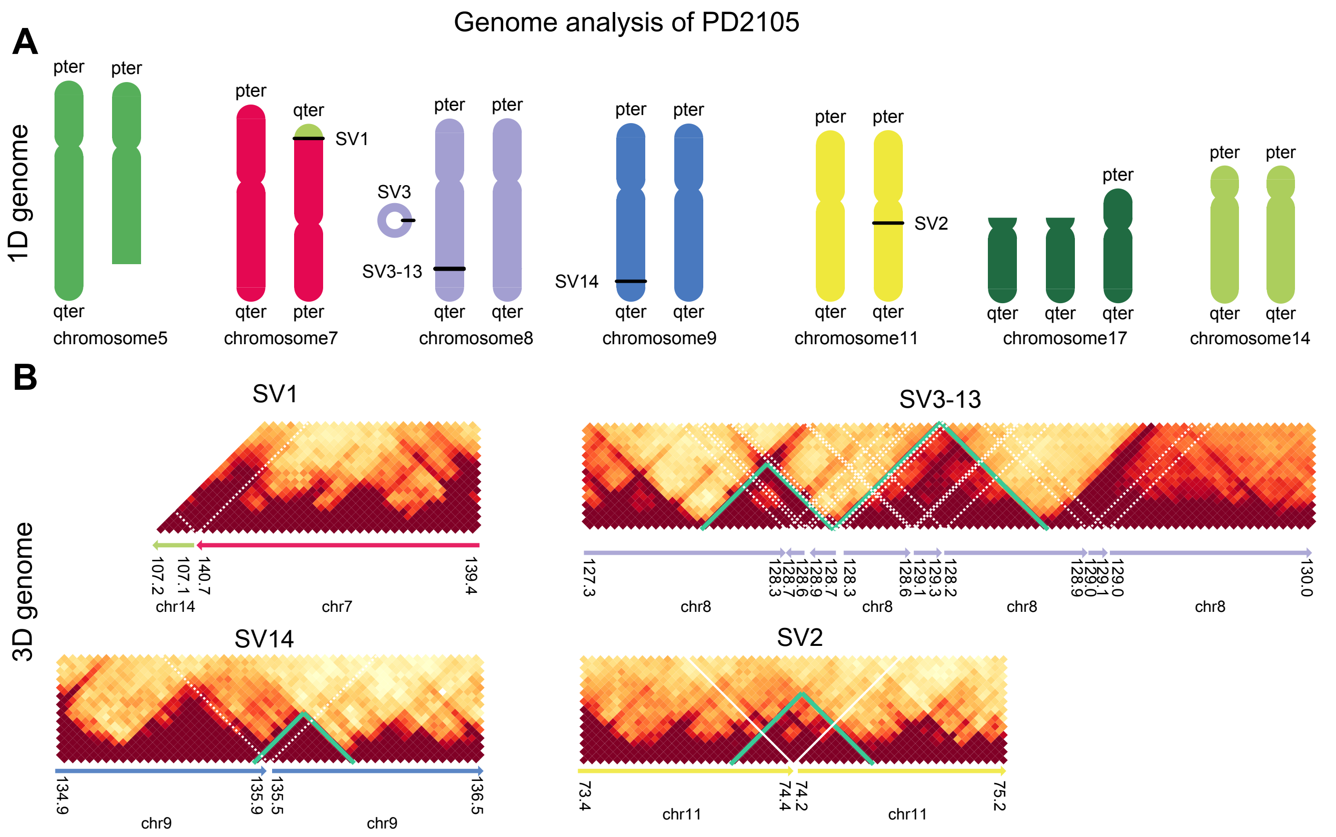

C

SV annotation

Index	Chr1	Pos1	Chr2	Pos2	Direction	Type	Coding	Non-coding
SV1	7	140725137	14	107060339	3to5	Tranlocation		
SV2	11	74212451	11	74375379	5to3	Duplication	Amplification of POLD3,RANP3	
SV3	8	128231851	8	129281861	5to3	Complex SV cluster (HSR/DM)	Amplification of MYC, POU5F1B	SE hijacking of MYC, POU5F1B
SV4	8	128883051	8	129134415	3to5			
SV5	8	128929953	8	129040523	3to5			
SV6	8	128251803	8	128656797	3to3			
SV7	8	128251846	8	128932032	5to5			
SV8	8	128579137	8	129012931	5to5			
SV9	8	128730194	8	128930061	5to5			
SV10	8	128864348	8	129040511	3to3			
SV11	8	128932004	8	128965032	3to3			
SV12	8	128579132	8	128867900	3to5			
SV13	8	128965035	8	129134305	5to3			
SV14	9	135465324	9	135889405	5to3	Duplication	Duplication of GTF3C4,DDX31,AK8, TSC1	SE hijacking of GFI1B

Appendix Figure S10. 1D genome and 3D genome analysis of the PD2105 patient. (**A**) SVs (SV1-14) are annotated in karyotypes (1D genome) of the PD2105 patient. (**B**) InfoHiC predicted the 3D genome from the 1D genome and found neo-TADs (green) resulted from SVs that are represented by dotted white lines. Reference coordinates are shown at the megabase scale under the contig Hi-C matrices. (**C**) SVs are classifed into simple and complex types, and their coding and non-coding effects are summarized in the SV annotation table.

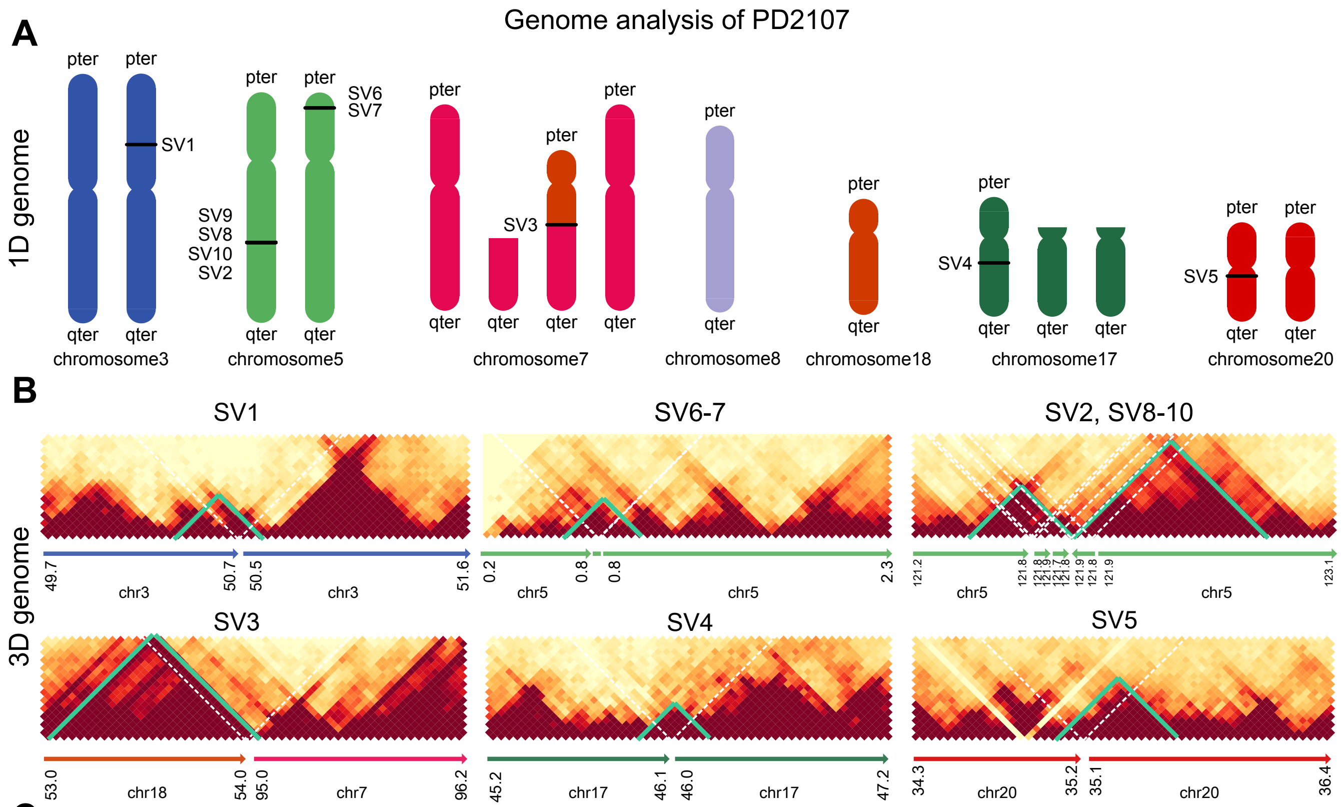

C

SV annotation

Index	Chr1	Pos1	Chr2	Pos2	Direction	Type	Coding	Non-coding
SV1	3	50480341	3	50696608	5to3	Duplication	Amplification of C3orf18,HEMK1, CISH, and MAPKAPK3	
SV3	7	95044286	18	54010505	5to3	Unbalanced translocation		
SV4	17	46005379	17	46144955	5to3	Duplication	Amplification of COPZ2,NFE2L1, PNPO, CDK5RAP3,PRR15L, MIR152, and NFE2L1-DT	
SV5	20	35080513	20	35190516	5to3	Duplication	Amplification of DLGAP4,MYL9, and DLGAP4-AS1	
SV6	5	701050	5	792312	3to5	Insertion		
SV7	5	696893	5	788253	5to3			
SV2	5	121670510	5	121857025	5to3	Complex SV cluster	Amplification of SNCAIP	Super-enhancer hijacking of PRDM6
SV8	5	121755843	5	121858045	5to5			
SV9	5	121755771	5	121858640	3to3			
SV10	5	121755150	5	121774504	5to3			

Appendix Figure S11. 1D genome and 3D genome analysis of the PD2107 patient. (**A**) SVs (SV1-10) are annotated in karyotypes (1D genome) of the PD2107 patient. (**B**) InfoHiC predicted the 3D genome from the 1D genome and found neo-TADs (green) resulted from SVs that are represented by dotted white lines. Reference coordinates are shown at the megabase scale under the contig Hi-C matrices. (**C**) SVs are classifed into simple and complex types, and their coding and non-coding effects are summarized in the SV annotation table.

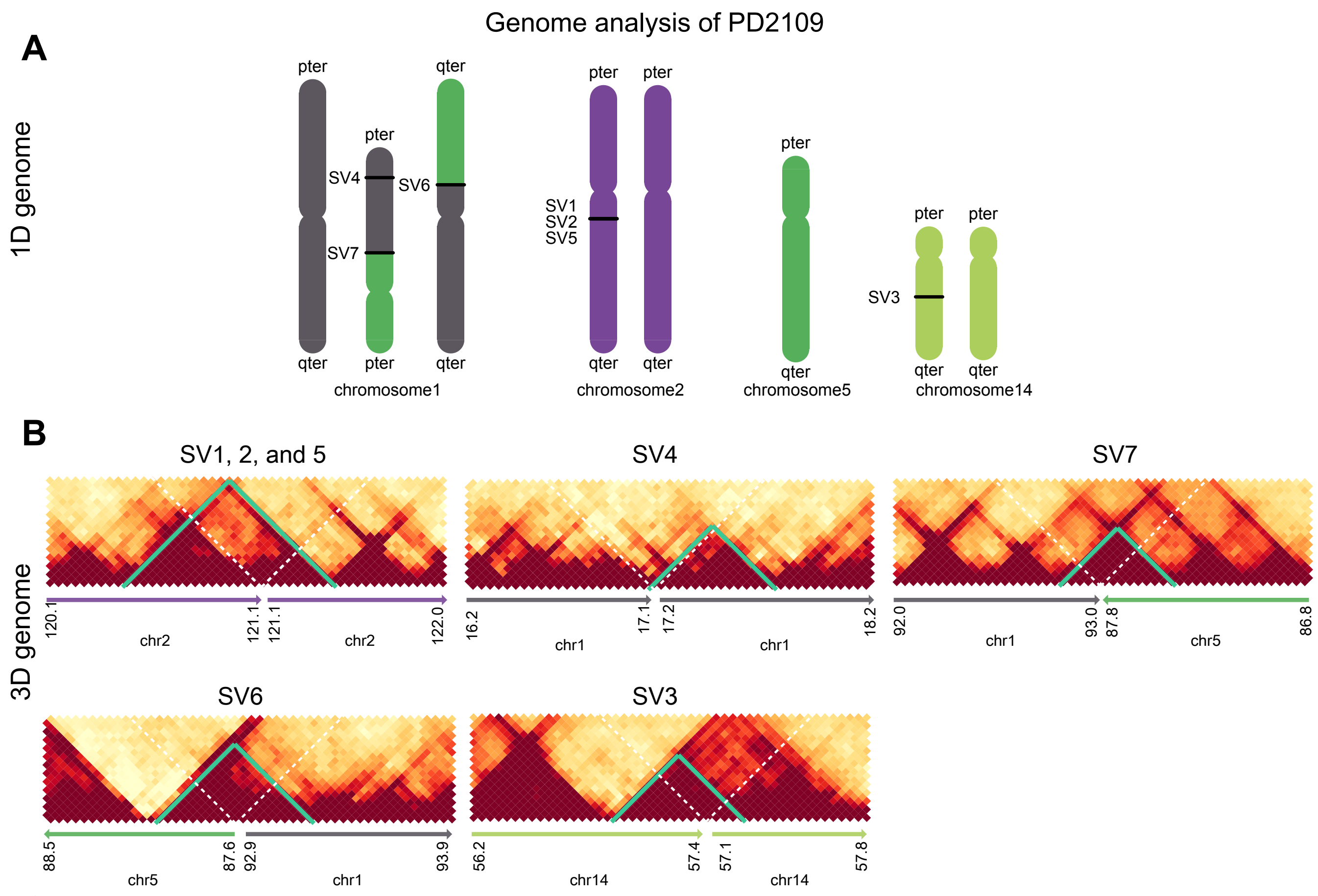

C

SV annotation

Index	Chr1	Pos1	Chr2	Pos2	Direction	Type	Coding	Non-coding
SV1	2	121100120	2	121102933	5to5	Complex SV cluster		
SV2	2	121100344	2	121103431	3to3			
SV5	2	121102544	2	121102940	5to5			
SV6	1	92901932	5	87599726	5to5	Reciprocal translocation		SE hijacking of EVI5,GFI1
SV7	1	92960263	5	87791087	3to3	Reciprocal translocation		SE hijacking of RPAP2,GFI1,GLMN
SV3	14	57069671	14	57401469	5to3	Duplication	Amplification of TMEM260,OTX2	
SV4	1	17054648	1	17189545	3to5	Deletion	Deletion of MST1L	

Appendix Figure S12. 1D genome and 3D genome analysis of the PD2109 patient. (**A**) SVs (SV1-7) are annotated in karyotypes (1D genome) of the PD2109 patient. (**B**) InfoHiC predicted the 3D genome from the 1D genome and found neo-TADs (green) resulted from SVs that are represented by dotted white lines. Reference coordinates are shown at the megabase scale under the contig Hi-C matrices. (**C**) SVs are classifed into simple and complex types, and their coding and non-coding effects are summarized in the SV annotation table.

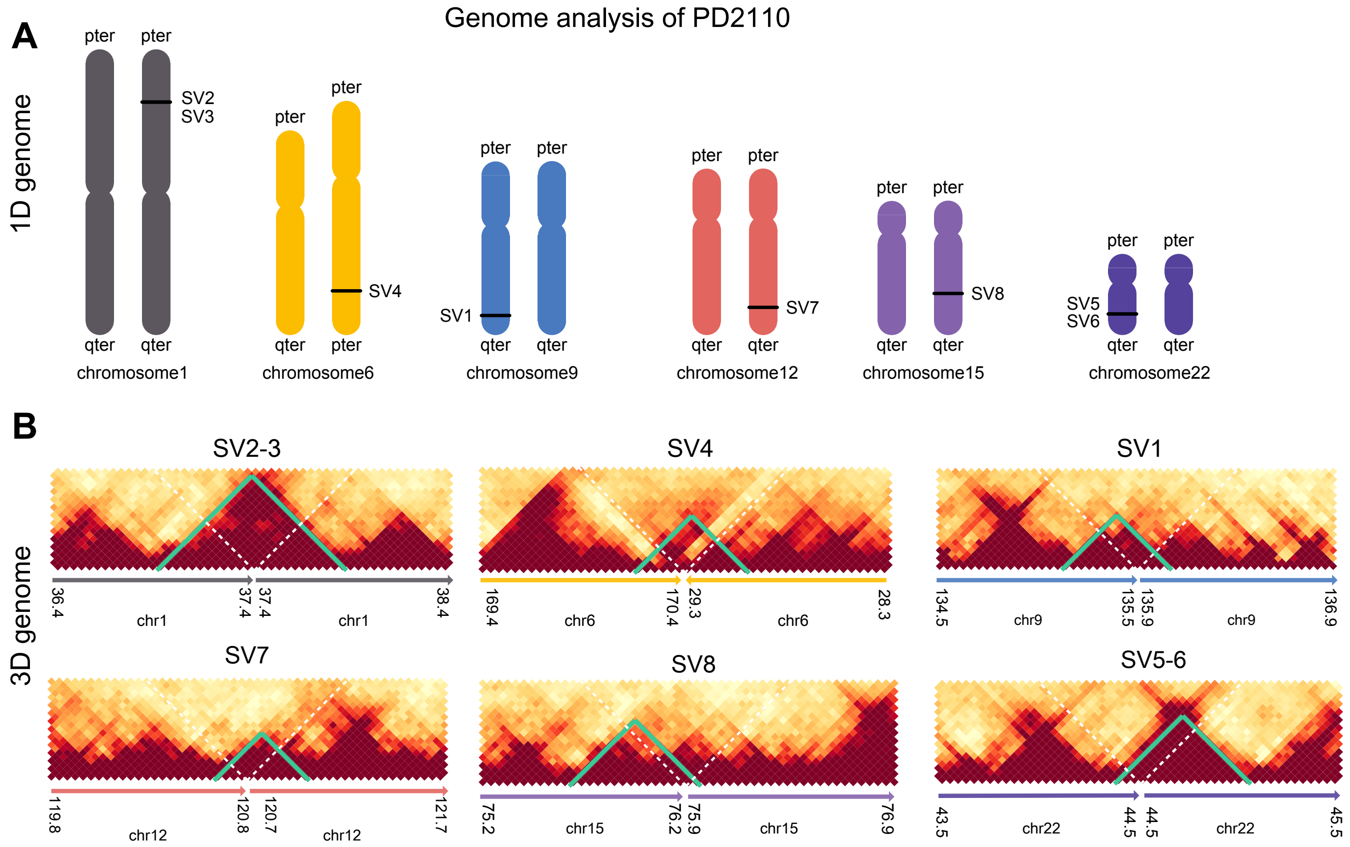

C

SV annotation

Index	Chr1	Pos1	Chr2	Pos2	Direction	Type	Coding	Non-coding
SV1	9	135515011	9	135853084	3to5	Deletion	Deletion of GTF3C4,DDX31,AK8, TSC1	SE hijacking of SETX,TTF1,DDX31, BARHL1,GTF3C5, RALGDS,CEL, GFI1B
SV2	1	37423057	1	37425492	3to5	Insertion		
SV3	1	37423210	1	37425631	5to3	Insertion		
SV4	6	29290776	6	170356220	3to3	Complex SV		
SV5	22	44510365	22	44511823	3to5	Insertion		
SV6	22	44511438	22	44511863	5to3	Insertion		
SV7	12	120705648	12	120819844	5to3	Duplication	Amplification of SIRT4,MSI1,PLA2G1B	
SV8	15	75899998	15	76189748	5to3	Duplication	Amplification of UBE2Q2,SNUPN,CSPG4, SNX33,IMP3,ODF3L1	

Appendix Figure S13. 1D genome and 3D genome analysis of the PD2110 patient. (**A**) SVs (SV1-8) are annotated in karyotypes (1D genome) of the PD2110 patient. (**B**) InfoHiC predicted the 3D genome from the 1D genome and found neo-TADs (green) resulted from SVs that are represented by dotted white lines. Reference coordinates are shown at the megabase scale under the contig Hi-C matrices. (**C**) SVs are classifed into simple and complex types, and their coding and non-coding effects are summarized in the SV annotation table.

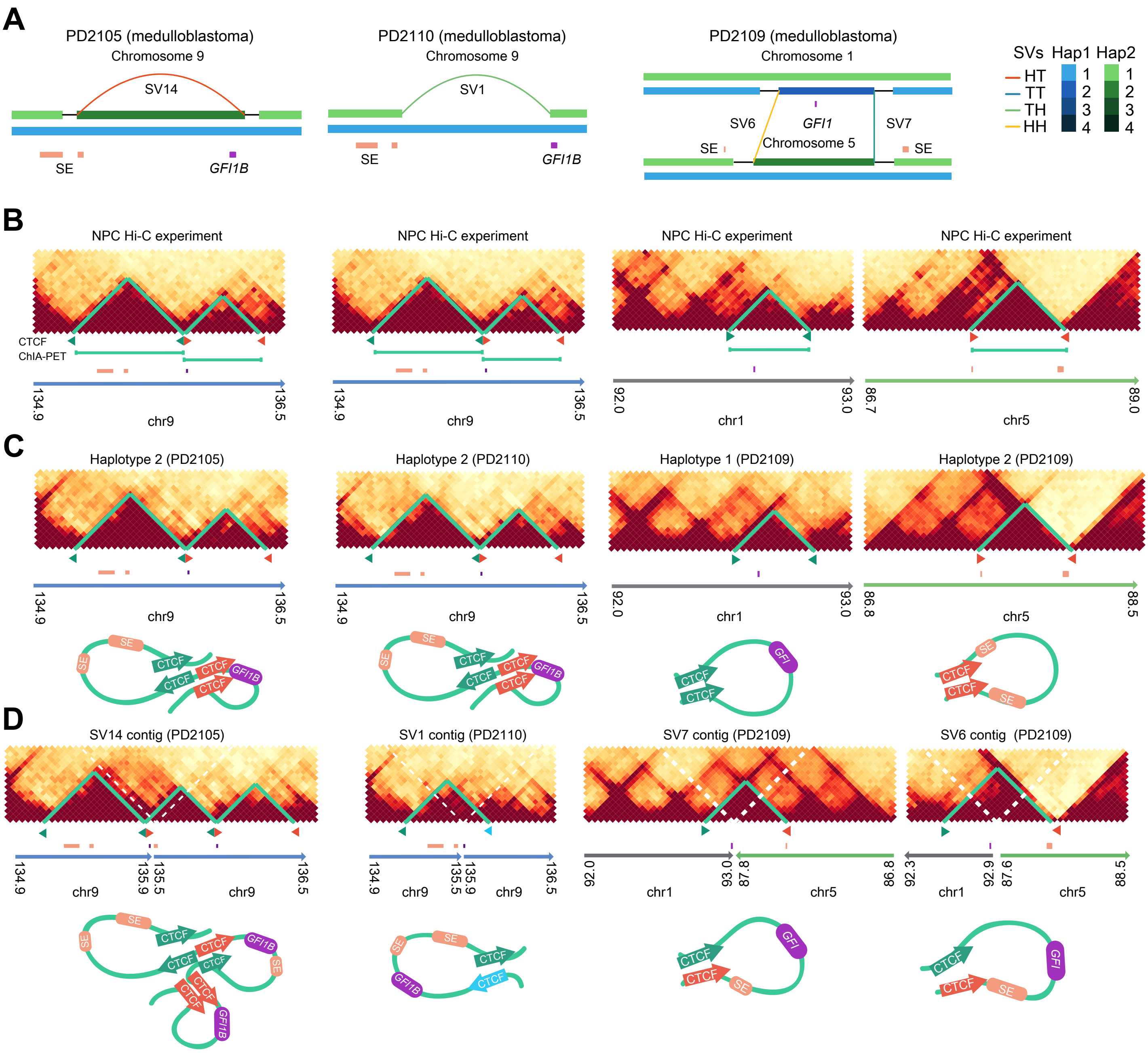

Appendix Figure S14. SE hijacking events of *GFI1* gene families in patients with medulloblastoma. (**A**) Haplotype graphs of the patients with medulloblastoma and (**B**) Hi-C experiments of the corresponding regions in neural progenitor cells (NPCs) are shown. Hi-C matrices of contigs without SVs (**C**) or with SVs (**D**) were predicted by InfoHiC. Reference TADs and neo-TADs are annotated (green) with CTCF motifs (arrowheads) near the TAD boundaries, and *GFI1B* and *GFI1* (purple) and SE (apricot) are annotated below. The pairwise interactions of the CTCF ChiA-PET data are indicated by green lines. 3D genome folding structures were modeled based on TAD boundaries and CTCF orientations.

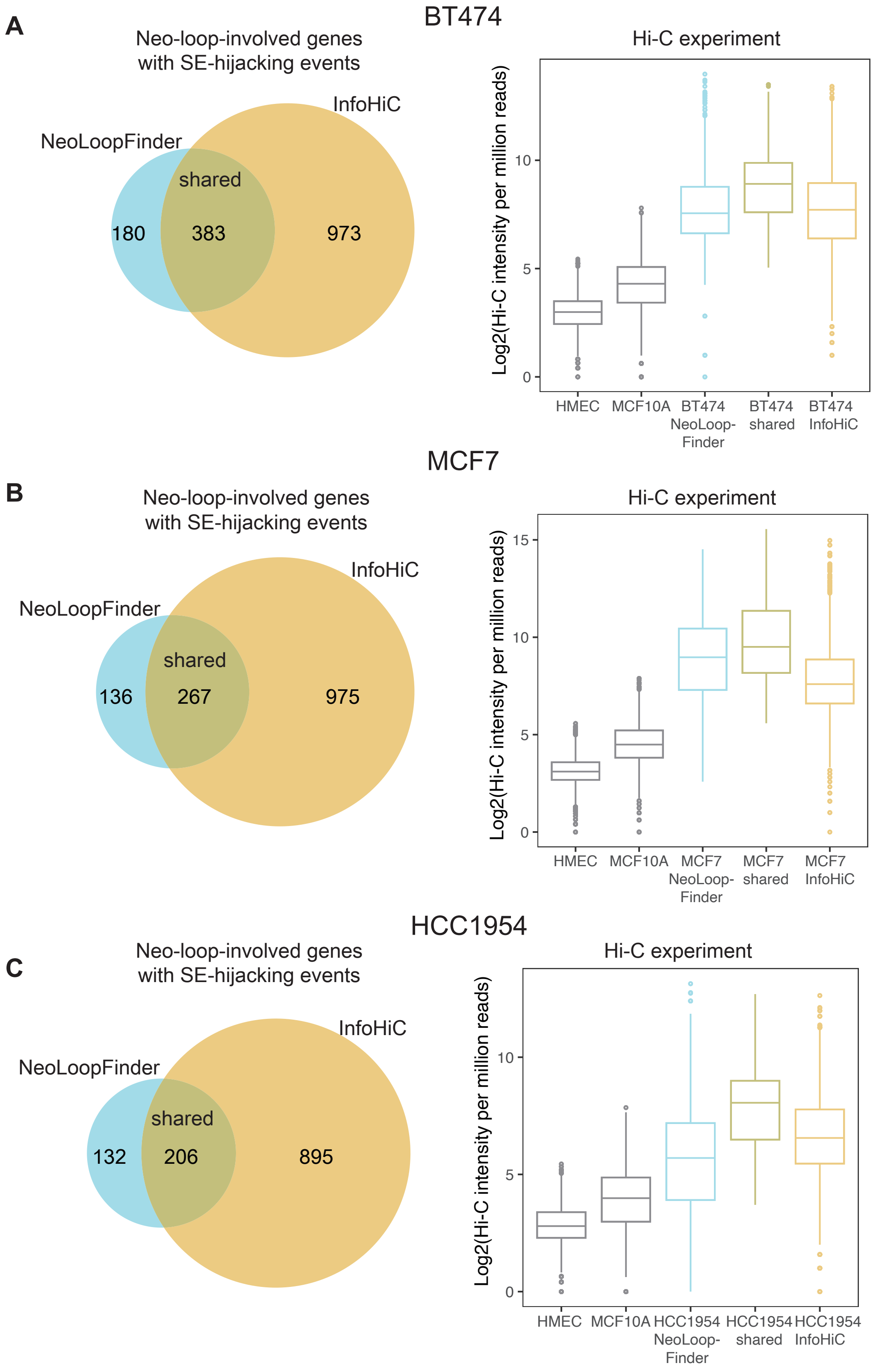
A
BT474
Neo-loop-involved genes
with SE-hijacking events
InfoHiC
NeoLoopFinder
shared
180
383
973
Hi-C experiment
Log2(Hi-C intensity per million reads)
0
5
10
HMEC
MCF10A
BT474 NeoLoop-Finder
BT474 shared
BT474 InfoHiC
MCF7
B
Neo-loop-involved genes
with SE-hijacking events
InfoHiC
NeoLoopFinder
shared
136
267
975
Hi-C experiment
Log2(Hi-C intensity per million reads)
0
5
10
15
HMEC
MCF10A
MCF7 NeoLoop-Finder
MCF7 shared
MCF7 InfoHiC
HCC1954
C
Neo-loop-involved genes
with SE-hijacking events
InfoHiC
NeoLoopFinder
shared
132
206
895
Hi-C experiment
Log2(Hi-C intensity per million reads)
0
5
10
HMEC
MCF10A
HCC1954 NeoLoop-Finder
HCC1954 shared
HCC1954 InfoHiC

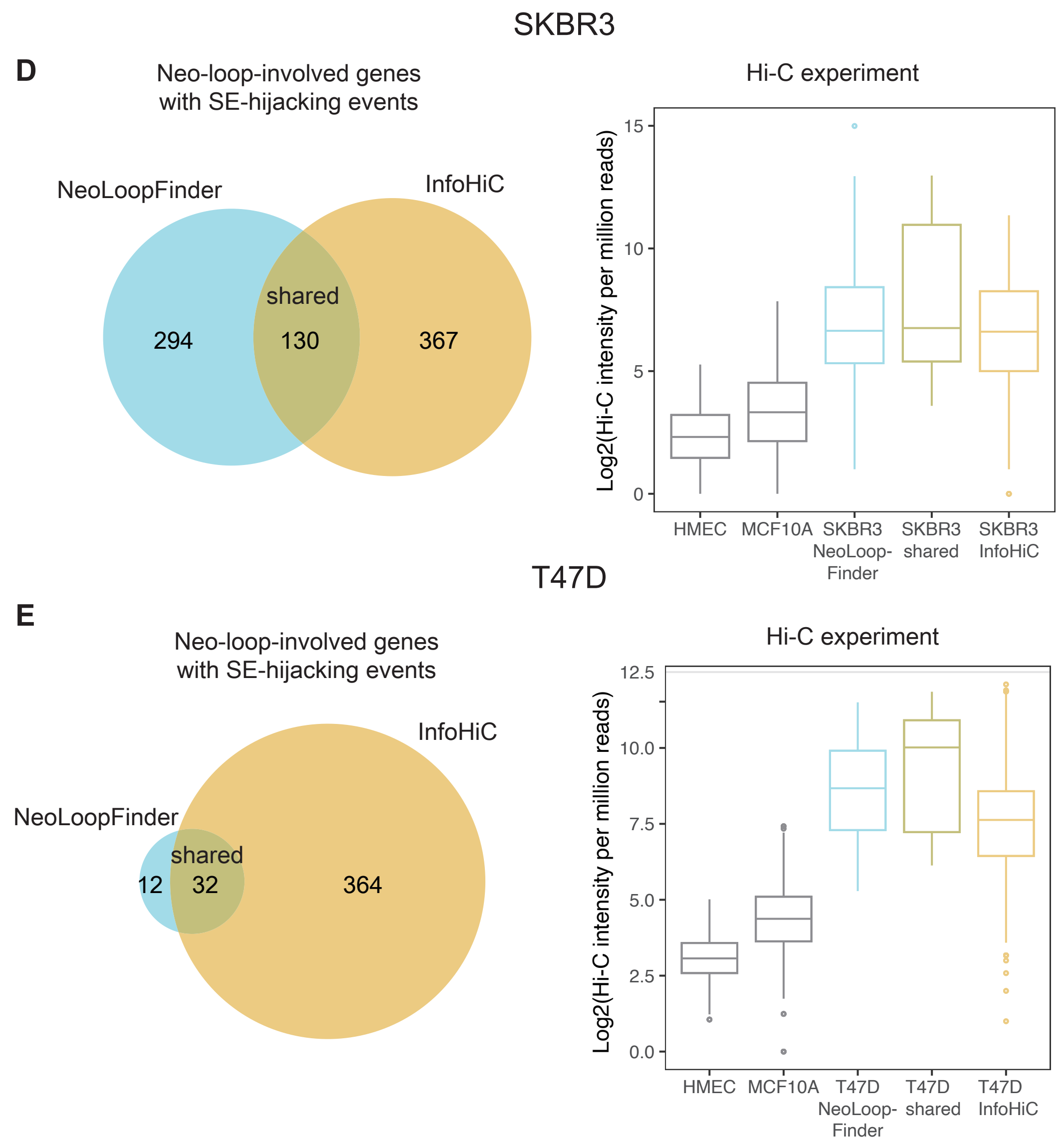

Appendix Figure S15. Comparison of neo-loop-involved genes found by NeoLoopFinder and InfoHiC.. A Venn diagram (left) of neo-loop-involved genes with SE hijacking events and Hi-C intensities in the Hi-C experiment (right) for each cancer cell line. Cancer cell lines include (**A**) BT474, (**B**) HCC1954, (**C**) MCF7, (**D**) SKBR3, and (**E**) T47D. The Venn diagram (left) shows portions of NeoLoopFinder-specific (cyan), InfoHiC-specific (orange), and shared events between them (brown). For each neo-loop-involved genes, Hi-C intensities of gene-SE pairs were measured. The box plot (right) shows Hi-C intensities of gene-SE pairs of neo-loop-involved genes observed in the cancer Hi-C experiment. Hi-C intensities of gene-SE pairs (either by NeoLoopFinder or InfoHiC, or shared) in HMEC and MCF10A experiments were measured as controls. The intensities were normalized as read counts per million Hi-C reads. Box plot colors represent events predicted by NeoLoopFinder (blue), InfoHiC (orange), and shared events between them (brown). Boxplot center lines are medians, box limits are upper and lower quartiles, and dots are outliers.

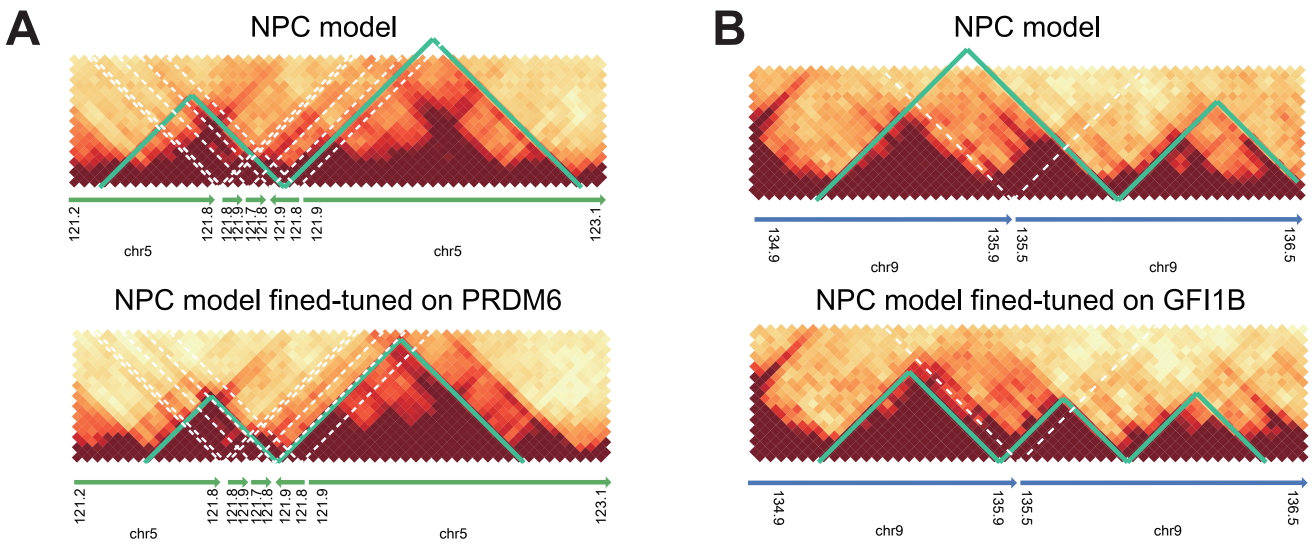

Appendix Figure S16. Prediction results using the fine-tuned models on medulloblastoma driver genes. Hi-C prediction results of the neo-TADs of *PRDM6* in the PD2107 patient (**A**), and *GFI1B* in the PD2105 patient (**B**). The NPC model fine-tuned on each gene (bottom) showed more accurate TAD annotation results (green) compared to the NPC model before fine-tuning (top).

Appendix Table S1. InfoHiC performance for reference windows of the test set 1.

	Test set 1 Reference window Prediction by T47D				
**Distance-stratified correlation (1Mb)**	**T47D**	**MCF7**	**BT474**	**SKBR3**	**HCC1954**
deepC	0.606769	0.593802	0.574972	0.580855	0.504865
InfoHiC(breakpoint_removal)	0.621986	0.59947	0.579731	0.587223	0.517852
InfoHiC(CSCN_decoding)	0.646703	0.626449	0.602341	0.606971	0.535348
InfoHiC(CSCN_encoding)	0.667845	0.640147	0.602586	0.622275	0.550141
deepC_2Mb	0.61597	0.595666	0.581352	0.587797	0.514311
InfoHiC_2Mb	0.666615	0.636142	0.610834	0.629562	0.548067
InfoHiC_transfer_2Mb	0.690626	0.666621	0.631422	0.648423	0.573594

Appendix Table S2. InfoHiC performance for reference windows and SV windows of the test set 2.

Test set 2

Reference window

Prediction by T47D

Measure	Methods	T47D	MCF7	BT474	SKBR3	HCC1954
**Distance stratified correlation (1Mb)**	deepC	0.602302	0.579349	0.513614	0.504287	0.471449
	InfoHiC(breakpoint_removal)	0.587662	0.571587	0.512853	0.508196	0.482222
	InfoHiC(CSCN_decoding)	0.663259	0.616618	0.657748	0.634476	0.532915
	InfoHiC(CSCN_encoding)	0.66078	0.615551	0.651963	0.634488	0.53113
	deepC_2Mb	0.625922	0.578872	0.520778	0.512774	0.485816
	InfoHiC_2Mb	0.668175	0.613179	0.643354	0.634573	0.539134
	InfoHiC_transfer_2Mb	0.686832	0.636323	0.664513	0.652946	0.559848

Reference window

Prediction by HMEC and MCF10A

Measure	Methods	T47D	MCF7	BT474	SKBR3	HCC1954
**Distance stratified correlation (1Mb)**	deepC_HMEC	0.554693	0.566463	0.517934	0.518682	0.512408
	deepC_HMEC_covNorm	0.601915	0.604004	0.573833	0.559613	0.559257
	InfoHiC_HMEC	0.6161	0.583927	0.639147	0.61874	0.529328
	deepC_MCF10A	0.582556	0.588163	0.53214	0.529738	0.47807
	deepC_MCF10A_covNorm	0.616087	0.613601	0.586142	0.563238	0.546563
	InfoHiC_MCF10A	0.635646	0.590915	0.654309	0.633169	0.502051
	deepC_HMEC_2Mb	0.561914	0.57231	0.529004	0.52909	0.515732
	InfoHiC_HMEC_2Mb	0.628226	0.580667	0.644853	0.629106	0.526822
	deepC_MCF10A_2Mb	0.592003	0.592231	0.543103	0.537028	0.4843
	InfoHiC_MCF10A_2Mb	0.644857	0.597216	0.658077	0.642096	0.516886

SV window

Prediction by T47D

Measure	Methods	T47D	MCF7	BT474	SKBR3	HCC1954
**Pearson correlation (1Mb)**	deepC-SV	0.648704	0.609156	0.6275	0.567585	0.548191
	InfoHiC	0.765478	0.73123	0.716717	0.680412	0.66747
**Pearson correlation (2Mb)**	deepC-SV_2Mb	0.701597	0.676915	0.657631	0.634732	0.599584
	InfoHiC_2Mb	0.754138	0.75745	0.719375	0.709989	0.674223

SV window

Prediction by HMEC and MCF10A

Measure	Methods	T47D	MCF7	BT474	SKBR3	HCC1954
**Pearson correlation (1Mb)**	deepC-SV_HMEC	0.697924	0.661657	0.666055	0.617538	0.609353
	deepC-SV_HMEC_covNorm	0.721133	0.714128	0.711381	0.66586	0.648625
	InfoHiC_HMEC	0.7415	0.72394	0.708668	0.675264	0.663478
	deepC-SV_MCF10A	0.706133	0.666479	0.672182	0.627666	0.606979
	deepC-SV_MCF10A_covNorm	0.722122	0.720394	0.709582	0.665901	0.641525
	InfoHiC_MCF10A	0.743637	0.727539	0.714219	0.676347	0.660204
**Pearson correlation (2Mb)**	deepC-SV_HMEC_2Mb	0.715132	0.688598	0.669005	0.646339	0.619784
	InfoHiC_HMEC_2Mb	0.750249	0.756472	0.712958	0.70404	0.670632
	deepC-SV_MCF10A_2Mb	0.716825	0.692934	0.675719	0.654161	0.617659
	InfoHiC_MCF10A_2Mb	0.749444	0.75906	0.720021	0.705849	0.668681

Appendix Table S3. Cancer-related genes of Neo-TADs found in breast cancer cell lines.

Sample_ID	Neo-TAD class	Gene
BT474	SE hijacking	GNA13
BT474	SE hijacking	ERBB2
BT474	TE hijacking	PTPRC
BT474	TE hijacking	SF1
BT474	TE hijacking	CCND1
BT474	TE hijacking	SPOP
BT474	TE hijacking	KAT7
BT474	TE hijacking	CACNA1A
BT474	TE hijacking	NFATC2
BT474	TE hijacking	PLAG1
BT474	TE hijacking	DMD
BT474	neo-TAD	BCL11B
BT474	neo-TAD	BRD4
BT474	neo-TAD	TRIM24
BT474	neo-TAD	TAL2
SKBR3	SE hijacking	MYC
SKBR3	SE hijacking	STAT3
SKBR3	SE hijacking	STAT5B
SKBR3	SE hijacking	GNAS
SKBR3	SE hijacking	BCL6
SKBR3	SE hijacking	HMGA1
SKBR3	SE hijacking	ATF1
SKBR3	TE hijacking	ERBB2
SKBR3	TE hijacking	PRKAR1A
SKBR3	TE hijacking	MECOM
SKBR3	TE hijacking	MUC4
SKBR3	TE hijacking	CNBD1
SKBR3	neo-TAD	CDH1
HCC1954	SE hijacking	TERT
HCC1954	SE hijacking	FGFR4
HCC1954	TE hijacking	GNAS
HCC1954	TE hijacking	PIK3CA
HCC1954	TE hijacking	FGFR3
HCC1954	TE hijacking	FBXW7
HCC1954	TE hijacking	HMGA1
HCC1954	TE hijacking	PSIP1
HCC1954	neo-TAD	FCRL4
HCC1954	neo-TAD	CDK4
HCC1954	neo-TAD	ERBB2
HCC1954	neo-TAD	EP300

Sample_ID	Neo-TAD class	Gene
MCF7	SE hijacking	ATP1A1
MCF7	SE hijacking	CHD4
MCF7	SE hijacking	MSI2
MCF7	SE hijacking	NFATC2
MCF7	SE hijacking	SALL4
MCF7	TE hijacking	NOTCH2
MCF7	TE hijacking	NRAS
MCF7	TE hijacking	FCRL4
MCF7	TE hijacking	ABL2
MCF7	TE hijacking	PAX3
MCF7	TE hijacking	CTNNB1
MCF7	neo-TAD	JAK1
MCF7	neo-TAD	PTPRC
MCF7	neo-TAD	DDX5
MCF7	neo-TAD	JAK3
MCF7	neo-TAD	CRTC1
MCF7	neo-TAD	MAPK1
MCF7	neo-TAD	TBL1XR1
MCF7	neo-TAD	MUC4
T47D	SE hijacking	NT5C2
T47D	TE hijacking	TERT
T47D	TE hijacking	TLX3
T47D	TE hijacking	NPM1

Appendix Table S4. Gene expression in neo-TADs and neo-loops in breast cancer cell lines.

Cancer cell line	Class	Group 1	Group 2	Group 1 N	Group 2 N	Group 1 median (FPKM)	Group 2 median (FPKM)	One-sided Student's t-test p-value
BT474	TAD	Reference	Neo TAD	9594	514	2.945365	3.656088	6.428E-15
BT474	TAD	Reference	SE hijacking	9594	100	2.945365	4.04752	3.787E-05
HCC1954	TAD	Reference	Neo TAD	10401	315	2.95097	3.181145	5.796E-03
HCC1954	TAD	Reference	SE hijacking	10401	75	2.95097	3.520837	1.201E-01
MCF7	TAD	Reference	Neo TAD	13333	435	2.781777	3.012064	2.904E-02
MCF7	TAD	Reference	SE hijacking	13333	104	2.781777	3.498776	1.233E-03
SKBR3	TAD	Reference	Neo TAD	12752	425	2.80557	3.060525	1.218E-03
SKBR3	TAD	Reference	SE hijacking	12752	192	2.80557	3.72802	2.653E-07
T47D	TAD	Reference	Neo TAD	9691	46	2.887451	2.884742	3.055E-01
T47D	TAD	Reference	SE hijacking	9691	45	2.887451	3.237954	4.280E-01
BT474	Loop	Reference	Neo loop	9537	608	2.835136	3.271407	1.206E-06
BT474	Loop	Reference	SE hijacking	9537	441	2.835136	3.63812	1.484E-14
HCC1954	Loop	Reference	Neo loop	9944	524	2.931289	3.310524	8.720E-05
HCC1954	Loop	Reference	SE hijacking	9944	323	2.931289	3.281637	6.055E-04
MCF7	Loop	Reference	Neo loop	12889	556	2.771938	2.89025	3.913E-01
MCF7	Loop	Reference	SE hijacking	12889	427	2.771938	3.38447	8.303E-08
SKBR3	Loop	Reference	Neo loop	12985	274	2.813968	3.154293	1.785E-03
SKBR3	Loop	Reference	SE hijacking	12985	104	2.813968	3.246656	2.487E-02
T47D	Loop	Reference	Neo loop	9524	186	2.88467	2.92709	2.791E-01
T47D	Loop	Reference	SE hijacking	9524	72	2.88467	3.393693	1.136E-01

Appendix Table S5. DeepC-SV specific cancer related genes involved with SE hijacking events and their Hi-C intensities.

Gene	Super enhancer (SEdb)	Cancer cell line	Hi-C intensity corresponding cancer cell line (left)	Hi-C intensity noncancerous cell line (MCF10A)	Hi-C intensity noncancerous cell cell line (HMEC)
CCND1	SE_02_05600663	BT474	0	0	0
CCND1	SE_02_05700895	BT474	0	0	0
SALL4	SE_01_07200274	BT474	0	0	0
MUC4	SE_01_07200856	SKBR3	28	23	9
MUC4	SE_02_05700781	SKBR3	28	22	19
MUC4	SE_01_07200856	MCF7	30	23	9
MUC4	SE_02_05700781	MCF7	31	22	19
CNBD1	SE_02_11500036	SKBR3	0	0	0
NRAS	SE_01_04600226	MCF7	3	0	0
JAK3	SE_02_05600655	MCF7	13	2	6
JAK3	SE_02_05600875	MCF7	36	13	19
JAK3	SE_02_07900144	MCF7	28	14	6
JAK3	SE_02_40300145	MCF7	28	14	6
PPM1D	SE_01_04501060	MCF7	2	0	0
PPM1D	SE_01_07200858	MCF7	4	0	0
TERT	SE_02_40100594	T47D	3	1	0
U2AF1	SE_01_04501149	HCC1954	3	6	4
U2AF1	SE_01_07200506	HCC1954	2	4	2
U2AF1	SE_02_05600338	HCC1954	3	6	4
U2AF1	SE_02_05700934	HCC1954	3	6	4
U2AF1	SE_02_39400338	HCC1954	29	0	0
SOX2	SE_01_04500829	HCC1954	12	0	0
SOX2	SE_02_05600722	HCC1954	12	0	0
SOX2	SE_02_05700975	HCC1954	12	0	0
SOX2	SE_02_40300315	HCC1954	12	0	0
BCL6	SE_02_05701179	HCC1954	24	4	4

Appendix Table S6. InfoHiC-specific SE hijacking events of cancer related genes and their Hi-C intensities.

Gene	Super enhancer (SEdb)	Cancer cell line	Hi-C intensity corresponding cancer cell line (left)	Hi-C intensity noncancerous cell line (MCF10A)	Hi-C intensity noncancerous cell cell line (HMEC)
TERT	SE_02_05701011	HCC1954	26	0	0
TERT	SE_02_40100474	HCC1954	11	0	0
GNAS	SE_02_39900298	SKBR3	148	2	3
BCL6	SE_02_07900084	SKBR3	103	8	7
BCL6	SE_02_39400161	SKBR3	103	8	7
BCL6	SE_02_40300180	SKBR3	103	8	7
NFATC2	SE_01_04600029	MCF7	19	7	8
NFATC2	SE_01_04600050	MCF7	55	12	11
NFATC2	SE_02_11500018	MCF7	46	13	17
SALL4	SE_01_04500251	MCF7	40	28	11
SALL4	SE_01_04600029	MCF7	35	0	0
SALL4	SE_01_04600050	MCF7	48	1	3
SALL4	SE_02_05600558	MCF7	40	28	11
SALL4	SE_02_05700122	MCF7	58	37	24
SALL4	SE_02_11500018	MCF7	85	2	0
NT5C2	SE_01_04500772	T47D	66	0	3
NT5C2	SE_01_07200072	T47D	142	2	8
NT5C2	SE_02_05600534	T47D	66	0	3
NT5C2	SE_02_05600945	T47D	43	0	4
NT5C2	SE_02_39400009	T47D	31	1	1
NT5C2	SE_02_39400010	T47D	26	1	1

Appendix Table S7. Gene expression in neo-TADs and neo-loops in BRCA datasets.

Copy number	Class	Group 1	Group 2	Group 1 N	Group 2 N	Group 1 median (FPKM-UQ)	Group 2 median (FPKM-UQ)	One-sided Student's t-test p-value
2	TAD	Reference	Neo TAD	900669	4363	2032.055	5469.532	2.505E-02
2	TAD	Reference	SE hijacking	900669	1434	2032.055	20142.872	3.293E-03
3	TAD	Reference	Neo TAD	835576	7364	2237.747	5969.908	4.937E-03
3	TAD	Reference	SE hijacking	835576	2480	2237.747	20495.543	8.730E-05
4	TAD	Reference	Neo TAD	746955	9816	2440.301	7525.502	1.410E-05
4	TAD	Reference	SE hijacking	746955	3023	2440.301	14139.272	4.662E-03
5	TAD	Reference	Neo TAD	332513	7775	3092.426	6215.572	4.590E-02
5	TAD	Reference	SE hijacking	332513	3093	3092.426	18560.642	2.508E-05
6	TAD	Reference	Neo TAD	111171	5535	3797.113	7892.995	4.603E-02
6	TAD	Reference	SE hijacking	111171	1904	3797.113	22785.099	9.080E-03
7	TAD	Reference	Neo TAD	52380	3136	3541.239	6716.476	1.454E-01
7	TAD	Reference	SE hijacking	52380	1332	3541.239	17572.565	1.509E-02
8	TAD	Reference	Neo TAD	17593	1839	4682.417	16460.727	5.804E-02
8	TAD	Reference	SE hijacking	17593	1012	4682.417	37017.548	1.664E-03
2	Loop	Reference	Neo loop	860538	10908	2022.828	3658.532	3.693E-01
2	Loop	Reference	SE hijacking	860538	5742	2022.828	16599.185	5.008E-06
3	Loop	Reference	Neo loop	852035	15608	2159.043	3961.954	4.479E-03
3	Loop	Reference	SE hijacking	852035	9072	2159.043	19259.917	1.116E-08
4	Loop	Reference	Neo loop	756233	20563	2364.223	4078.969	1.698E-02
4	Loop	Reference	SE hijacking	756233	11898	2364.223	24092.065	2.195E-16
5	Loop	Reference	Neo loop	326213	15003	2967.299	4071.06	1.134E-01
5	Loop	Reference	SE hijacking	326213	8856	2967.299	22864.683	1.795E-06
6	Loop	Reference	Neo loop	104949	8818	3493.987	5495.944	3.665E-02
6	Loop	Reference	SE hijacking	104949	6434	3493.987	29350.803	5.625E-05
7	Loop	Reference	Neo loop	48319	4837	3209.894	4927.636	3.954E-01
7	Loop	Reference	SE hijacking	48319	3692	3209.894	28150.983	1.122E-05
8	Loop	Reference	Neo loop	15357	2648	3965.245	9519.49	4.956E-02
8	Loop	Reference	SE hijacking	15357	2439	3965.245	42230.858	1.513E-05

Appendix Table S8. Recurrent neo-TAD and enhancer hijacking genes with overexpression.

Gene symbol	Total samples with neo TAD	Neo TAD	Enhancer hijacking	Super enhancer hijacking	Neo TADs Cook's distance >n/4	Enhancer hijacking Cook's distance >n/4	Super enhancer hijacking Cook's distance >n/4	FPKM UQ thres	Log-rank survival p-value	BH corrected p-value
LASP1	7	1	1	5	0	1	4	3E+06	0.0011	0.0193
CLTC	6	0	1	5	0	1	4	3E+06	0.0048	0.0432
MYO1D	7	1	0	6	1	0	5	700000	0.0073	0.0435
INTS2	6	0	1	5	0	1	4	150000	0.0402	0.1811
EFCAB3	7	0	3	4	0	2	4	400	0.0685	0.2464
DHX40	7	0	2	5	0	1	4	400000	0.1599	0.4209
CDK12	15	1	4	10	1	3	5	250000	0.1637	0.4209
MIR4728	19	2	4	13	0	4	10	25000	0.2448	0.5367
PSMD3	13	0	3	10	0	2	8	2E+06	0.3118	0.5367
STARD3	18	2	3	13	0	3	12	350000	0.3121	0.5367
NEUROD2	16	2	3	11	1	3	7	600	0.3534	0.5367
MSL1	12	0	7	5	0	4	5	1E+06	0.3578	0.5367
ERBB2	19	2	4	13	0	3	9	2E+06	0.5301	0.6925
TCAP	18	2	3	13	0	3	10	25000	0.5439	0.6925
CCND1	11	2	3	6	0	2	5	4E+06	0.5771	0.6925
PTRH2	6	0	1	5	0	0	4	250000	0.8055	0.8693
GSDMB	16	1	0	15	1	0	9	150000	0.8210	0.8693
FBXL20	13	1	8	4	0	5	4	150000	0.8822	0.8822

Appendix Table S9. Exonic SNVs and indels found in patients with medulloblastoma.

Sample	Variant_information									Driver annotation				Population		RNA	
	Gene	Chr	Pos	Ref	Alt	Type	Ref	Alt	VAF	COSMIC cancer gene census	Cancer driver	MB driver	clinvar	dbSNP	gnomAD	Ref	Alt
PD2104	NOTCH2	1	120612002	CGG	C	frameshift	164	67	0.290	O			Benign/ Likely_benign	O		4	2
	CDH10	5	24488122	C	T	nonsynonymous	57	6	0.095	O						79	0
	BTK	X	100609666	A	T	stopgain	41	3	0.068	O						18	0
	KIAA2018	3	113376110	TTGCT GCTGC	T	nonframeshift	26	39	0.600				Benign	O		3	2
	MAML3	4	140811063	TTGC	T	frameshift	49	41	0.456					O	O	26	13
	NBPF11	1	146055349	G	A	nonsynonymous	41	4	0.089					O	O	97	0
	TUFT1	1	151534586	G	T	nonsynonymous	88	4	0.043							33	0
	FCGR3A	1	161518422	C	G	nonsynonymous	114	8	0.066					O	O	31	0
	RBM15B	3	51428840	C	A	nonsynonymous	86	4	0.044							32	0
	NKD2	5	1009180	A	C	nonsynonymous	49	8	0.140							15	0
	CLTB	5	175843196	G	A	nonsynonymous	94	4	0.041							584	0
	MUC22	6	30994980	T	C	nonsynonymous	49	6	0.109							0	0
	SP8	7	20824973	T	G	nonsynonymous	60	7	0.104							0	0
	POM121	7	72414028	A	G	nonsynonymous	143	7	0.047					O		223	0
	AKR1D1	7	137761269	A	G	nonsynonymous	92	4	0.042					O		0	0
	OR4F21	8	116247	G	A	nonsynonymous	3	7	0.700							2	0
	ERICH1	8	642595	G	C	nonsynonymous	37	16	0.302							19	7
	MAFA	8	144511981	T	G	nonsynonymous	34	5	0.128					O		0	0
	ANKRD20A4	9	69423796	A	G	nonsynonymous	83	4	0.046					O		6	0
	EIF5AL1	10	81272659	A	G	nonsynonymous	65	5	0.071					O		111	0
	BTAF1	10	93702222	G	C	nonsynonymous	55	25	0.313							54	12
	MUC5B	11	1266479	G	T	nonsynonymous	76	5	0.062					O	O	17	1
	OLFML1	11	7530944	C	A	nonsynonymous	60	11	0.155							5	0
	ZNF705E	11	71529855	G	A	stopgain	95	7	0.069							4	0
	OR11H2	14	20181357	A	C	nonsynonymous	94	7	0.069					O	O	0	0
	AHNAK2	14	105408755	G	C	nonsynonymous	75	7	0.085					O	O	4	0
	GOLGA6L1	15	22743596	C	T	stopgain	41	5	0.109					O	O	0	0
	PDE8A	15	85669604	A	G	nonsynonymous	66	4	0.057							23	0
	PDXDC1	16	15122780	G	C	nonsynonymous	91	4	0.042							227	20
	CDRT1	17	15518981	G	C	nonsynonymous	103	5	0.046					O		1	0
	ARL17A	17	44606164	G	A	nonsynonymous	118	5	0.041					O		83	0
	STRA13	17	79977215	G	A	nonsynonymous	164	6	0.035					O	O	324	1
	ANKRD12	18	9258883	C	A	stopgain	82	4	0.047							190	0
	CABLES1	18	20715751	A	C	nonsynonymous	40	3	0.070							0	0
	SIGLEC15	18	43417835	G	A	nonsynonymous	137	5	0.035							3	0
	MBD2	18	51750487	T	G	nonsynonymous	33	9	0.214							32	0
	ZNF254	19	24309874	G	T	nonsynonymous	65	8	0.110							81	2
	RFPL4A	19	56274213	T	A	nonsynonymous	81	7	0.080					O		0	0
	TFF1	21	43786574	C	A	nonsynonymous	66	4	0.057					O	O	0	0
	POTEH	22	16277757	C	T	nonsynonymous	59	5	0.078					O		0	0
	GGA1	22	38016901	A	G	nonsynonymous	79	4	0.048							252	0
	XIAP	X	123040988	G	A	nonsynonymous	64	4	0.059							92	0
	ASCL1	12	103352207	A	AGCAG CAGCT	nonframeshift	82	31	0.274						O	0	0
	FMNL1	17	43319458	GCCT	G	nonframeshift	64	30	0.319							25	0

	MADCAM1	19	501682	G	GACCTCCCCGGAGCCTCCCAACACC	nonframeshift	57	28	0.329							0	0
PD2105	CTDNEP1	17	7149641	AG	A	frameshift	36	16	0.308			O				365	42
	MTOR	1	11298590	C	A	nonsynonymous	82	5	0.057	O	O					49	0
	ATRX	X	76777788	G	T	nonsynonymous	41	3	0.068	O	O					162	0
	NCOR2	12	124824739	T	TGCCG	frameshift	34	51	0.600	O					O	314	0
	OR2T33	1	248436638	A	G	nonsynonymous	96	5	0.050				Benign	O		0	0
	GOLGA6B	15	72954797	T	C	nonsynonymous	81	4	0.047					O	O	0	0
	IGLL1	22	23915574	G	A	nonsynonymous	83	5	0.057				Benign	O	O	3	0
	VCX3A	X	6451842	G	C	nonsynonymous	21	4	0.160				Benign/ Likely_benign	O		0	0
	MEF2A	15	100252709	CCAG	C	nonframeshift	44	31	0.413					O	O	124	0
	TAF4	20	60640305	TGCCAGG	T	nonframeshift	0	2	1.000				Benign	O	O	0	0
	ZSWIM6	5	60628574	CCGGCCGCAACCTCGG	C	nonframeshift	2	2	0.500				Benign			0	0
	MNX1	7	156802643	A	AGCGGCG	nonframeshift	11	2	0.154				Benign/ Likely_benign			0	0
	SMARCA2	9	2039776	ACAG	A	nonframeshift	42	65	0.607				Benign/ Likely_benign	O		42	10
	GJC2	1	228346502	A	C	nonsynonymous	59	9	0.132							8	0
	POTEE	2	132010612	G	A	nonsynonymous	91	7	0.071					O		2	0
	CCDC74A	2	132290441	G	A	nonsynonymous	105	7	0.063					O		27	0
	TMEM14B	6	10756728	C	T	nonsynonymous	93	4	0.041					O	O	417	0
	POM121	7	72412783	A	T	nonsynonymous	40	3	0.070					O	O	90	0
	POM121C	7	75051619	G	T	nonsynonymous	77	5	0.061					O	O	39	0
	MUC17	7	100683020	G	C	nonsynonymous	86	6	0.065					O		0	0
	FAM86B2	8	12291577	C	T	nonsynonymous	71	4	0.053							14	0
	COL22A1	8	139611003	G	A	nonsynonymous	61	34	0.358							14	0
	AGAP6	10	51748673	C	G	nonsynonymous	80	4	0.048					O		102	4
	STK32C	10	134121213	T	G	nonsynonymous	36	4	0.100							5	0
	MUC2	11	1093286	C	G	nonsynonymous	32	4	0.111					O	O	0	0
	TRIM64B	11	89608164	G	T	nonsynonymous	143	6	0.040							0	0
	SLC2A3	12	8074227	G	A	nonsynonymous	80	5	0.059							56	0
	DDX11	12	31244665	C	T	nonsynonymous	103	7	0.064					O		67	11
	HCAR3	12	123200158	G	C	nonsynonymous	103	6	0.055					O		0	0
	TRIP11	14	92471884	C	A	nonsynonymous	82	4	0.047							19	0
	AHNAK2	14	105413471	T	C	nonsynonymous	83	7	0.078					O		17	0
	WFDC1	16	84353055	C	T	nonsynonymous	65	37	0.363					O	O	48	3
	ZFPM1	16	88600189	A	C	nonsynonymous	28	4	0.125							3	1
	TVP23C	17	15449158	A	G	nonsynonymous	58	5	0.079					O		16	1
	KRTAP4-7	17	39240795	A	T	nonsynonymous	88	12	0.120					O		0	0
	KRTAP10-4	21	45994014	C	T	nonsynonymous	90	7	0.072					O	O	0	0
	KRTAP10-6	21	46012238	T	C	nonsynonymous	49	6	0.109							0	0
	CUL4B	X	119666278	G	A	nonsynonymous	7	28	0.800							88	97
	KRTAP5-5	11	1651199	A	AGGCTGTGGCTCC	nonframeshift	51	23	0.311					O		0	0
	ARMCX4	X	100749016	G	GAGGCTC	nonframeshift	26	31	0.544							0	0
	KMT2C	7	151935910	C	T	nonsynonymous	133	6	0.043	O	O			O		67	0
	OR2T34	1	248737734	G	A	nonsynonymous	81	7	0.080					O	O	0	0
	ALPPL2	2	233273011	C	G	nonsynonymous	76	11	0.126				Benign	O	O	0	0

PD2107	ZNF780A	19	40581109	T	C	nonsynonymous	58	6	0.094							20	0
	CELA1	12	51740414	GAC	G	frameshift	37	13	0.260				Benign	O		0	0
	CRIPAK	4	1388850	C	T	nonsynonymous	11	3	0.214					O	O	49	4
	TRPC7	5	135587516	A	G	nonsynonymous	50	4	0.074							0	0
	PCDHB13	5	140594149	A	G	nonsynonymous	75	4	0.051					O	O	181	0
	BCLAF1	6	136599822	C	G	nonsynonymous	80	4	0.048					O		113	0
	OR4F21	8	116636	A	C	nonsynonymous	22	4	0.154							0	0
	SPATA31C1	9	90536581	G	A	nonsynonymous	87	4	0.044					O		0	0
	SPATA31C1	9	90538028	C	T	nonsynonymous	68	4	0.056					O		0	0
	SPATA31C2	9	90749693	T	C	nonsynonymous	50	8	0.138					O	O	0	0
	NUTM2G	9	99697735	C	A	nonsynonymous	94	4	0.041					O		32	0
	AGAP7P	10	51464720	G	A	nonsynonymous	40	3	0.070					O		20	0
	GJA3	13	20716950	C	A	stopgain	83	4	0.046							1	0
	KL	13	33590735	G	T	nonsynonymous	34	3	0.081							0	0
	OR11H2	14	20181348	A	G	nonsynonymous	77	5	0.061					O		0	0
	GOLGA8J	15	30382038	A	G	nonsynonymous	45	4	0.082					O		4	0
	ZNF594	17	5087355	A	C	nonsynonymous	35	3	0.079							23	0
	DNAH2	17	7643253	A	C	nonsynonymous	31	3	0.088							6	0
	ARHGEF15	17	8215690	A	C	nonsynonymous	34	3	0.081							1	0
	TEKT3	17	15231383	C	A	stopgain	40	3	0.070							5	0
	CD209	19	7810724	A	T	nonsynonymous	50	6	0.107							0	0
	PLVAP	19	17476250	G	A	nonsynonymous	96	12	0.111					O		0	0
	ZNF90	19	20229964	C	A	nonsynonymous	63	8	0.113					O		14	0
	HKR1	19	37853452	C	A	nonsynonymous	33	32	0.492							86	60
	ZNF780A	19	40581115	C	A	nonsynonymous	55	7	0.113							22	0
	NSFL1C	20	1445040	C	T	nonsynonymous	75	4	0.051							299	0
	SHROOM2	X	9862955	A	C	nonsynonymous	41	5	0.109							3	0
	FAM47C	X	37027673	A	C	nonsynonymous	20	10	0.333							0	0
	MAOB	X	43628608	G	T	nonsynonymous	29	4	0.121							106	0
	DMRTB1	1	53925388	G	GCCCCCC	nonframeshift	28	14	0.333							0	0
	PPM1E	17	56833478	A	ACCCGAC	nonframeshift	70	91	0.565							28	0
	BPTF	17	65955782	A	AGCCCCACCC	nonframeshift	28	43	0.606							305	0
	GDF7	2	20867122	G	GGGCGGCGGC	nonframeshift	3	4	0.571						O	0	0
	ARMCX4	X	100749044	C	CTGAGGT	nonframeshift	29	19	0.396							0	0
	NCOR2	12	124824739	T	TGCCG	frameshift	39	69	0.639	O					O	403	0
	OR2T33	1	248436638	A	G	nonsynonymous	93	4	0.041				Benign	O		0	0
	TUBB2A	6	3154882	C	T	nonsynonymous	89	4	0.043				Benign	O		2913	0
	TAF4	20	60640305	TGCCAGG	T	nonframeshift	0	2	1.000				Benign	O	O	1	0
	BSDC1	1	32841997	C	T	nonsynonymous	56	36	0.391					O		177	135
	SLC5A9	1	48708204	G	A	nonsynonymous	65	27	0.293							0	0
	OR2L8	1	248112754	T	C	nonsynonymous	46	6	0.115					O		0	0
	GTF3C2	2	27552149	G	T	nonsynonymous	78	15	0.161							120	36
	TTN	2	179477286	G	A	nonsynonymous	71	8	0.101					O		17	0
	FAM157A	3	197896656	G	A	nonsynonymous	190	13	0.064					O	O	1	1
	UST	6	149068568	T	C	nonsynonymous	96	11	0.103							1	0
	MUC17	7	100685790	C	A	nonsynonymous	70	4	0.054							0	0
	ADAM28	8	24200633	G	C	nonsynonymous	69	4	0.055							2	0
	OR13C5	9	107361627	A	T	nonsynonymous	70	6	0.079					O	O	1	2
	AGAP6	10	51769578	C	T	nonsynonymous	83	4	0.046					O		163	0
	VENTX	10	135053527	C	T	nonsynonymous	73	8	0.099					O	O	2	0

	USH1C	11	17531108	A	C	nonsynonymous	35	9	0.205							161	0
	AHNAK	11	62290036	T	A	nonsynonymous	93	7	0.070							296	0
	AHNAK	11	62295933	T	C	nonsynonymous	57	9	0.136							154	12
	ZNF705E	11	71529854	T	C	nonsynonymous	83	4	0.046					O		0	0
	TRIM64B	11	89608966	A	C	nonsynonymous	46	5	0.098							0	0
PD2109	A2ML1	12	9027043	A	C	nonsynonymous	66	8	0.108							0	0
	PRB1	12	11506749	T	A	nonsynonymous	46	5	0.098					O		1	0
	KIAA1551	12	32136371	T	C	nonsynonymous	63	4	0.060							54	0
	KIAA0226L	13	46952042	C	T	nonsynonymous	82	4	0.047					O		1	0
	AHNAK2	14	105412633	G	A	nonsynonymous	55	8	0.127							33	0
	AHNAK2	14	105412658	G	C	nonsynonymous	67	12	0.152							32	0
	AHNAK2	14	105418124	C	G	nonsynonymous	75	4	0.051					O		13	0
	NPIPB6	16	28353929	T	C	stoploss	52	5	0.088					O	O	9	0
	LOC100129697	16	89017569	G	C	nonsynonymous	67	7	0.095					O	O	57	2
	SLFN13	17	33772558	G	A	nonsynonymous	63	8	0.113					O		0	0
	ZNF257	19	22271297	G	A	nonsynonymous	52	6	0.103					O		3	0
	ZNF98	19	22575777	T	C	nonsynonymous	72	7	0.089					O		3	0
	HPN	19	35556160	T	A	nonsynonymous	60	30	0.333							7	0
	TRPM4	19	49713994	G	A	nonsynonymous	66	36	0.353							20	10
	LILRB2	19	54778617	T	C	nonsynonymous	73	5	0.064					O	O	0	1
	B4GALT5	20	48330182	G	A	nonsynonymous	64	4	0.059							16	0
	SLC5A1	22	32480573	C	T	nonsynonymous	91	9	0.090					O	O	0	0
	RRP7A	22	42910199	G	A	nonsynonymous	99	6	0.057					O		109	3
	PARVB	22	44420301	A	C	nonsynonymous	38	7	0.156							29	0
	VCX3B	X	8434354	T	C	nonsynonymous	36	4	0.100					O	O	1	0
	CCNB3	X	50054188	T	G	nonsynonymous	39	3	0.071							21	0
	CTAG2	X	153881578	G	A	nonsynonymous	5	50	0.909					O	O	0	0
	ATAD3B	1	1420466	T	A	nonsynonymous	92	5	0.052				Likely benign	O	O	305	0
	GOLGA6L10	15	83014132	C	G	nonsynonymous	37	3	0.075							45	0
	SLC2A10	20	45354879	G	A	nonsynonymous	103	11	0.096				Uncertain significance	O	O	0	0
	KRTAP5-5	11	1651190	CGGCT GTGGA	C	nonframeshift	38	20	0.345						O	0	0
	NCOR2	12	124887093	C	CTGT	nonframeshift	33	59	0.641	O						257	0
	NEFH	22	29885622	A	AAGGA AG	nonframeshift	53	25	0.321				Uncertain significance			54	0
	KIAA0754	1	39879250	A	G	nonsynonymous	15	6	0.286					O	O	352	0
	NBPF12	1	146420149	G	A	nonsynonymous	36	3	0.077					O		13	0
	CENPL	1	173772127	A	T	nonsynonymous	81	4	0.047							19	0
	ALMS1	2	73717556	C	T	nonsynonymous	71	4	0.053							69	0
	POLR1A	2	86281406	A	T	nonsynonymous	94	4	0.041							43	0
	RGPD5	2	113127775	G	C	nonsynonymous	66	4	0.057					O	O	41	0
	DCAF17	2	172337598	G	T	stopgain	66	12	0.154							109	4
	SLC9B1	4	103827752	A	G	nonsynonymous	85	5	0.056					O		10	0
	SPATA9	5	95018546	G	A	nonsynonymous	56	28	0.333					O		1	1
PD2110	FAM153B	5	175528584	C	T	nonsynonymous	92	5	0.052					O	O	22	0
	MDC1	6	30673013	A	C	nonsynonymous	51	8	0.136					O	O	236	1
	ANKRD20A3	9	42368591	C	A	nonsynonymous	46	4	0.080							4	0
	ANKRD20A4	9	69424169	A	C	nonsynonymous	99	5	0.048							0	0
	OR13C5	9	107361627	A	T	nonsynonymous	74	5	0.063					O	O	4	1
	CAPZA3	12	18891826	C	A	nonsynonymous	67	4	0.056							0	0
	EFNB2	13	107145632	C	T	nonsynonymous	77	4	0.049					O		11	0
	GOLGA6L4	15	84908878	T	C	nonsynonymous	24	3	0.111					O	O	33	2
	TPSAB1	16	1290947	A	G	nonsynonymous	69	5	0.068					O		9	0

	KLHDC4	16	87760408	T	G	nonsynonymous	34	10	0.227							75	0
	ZNF98	19	22575777	T	C	nonsynonymous	53	5	0.086					O		0	0
	ZNF98	19	22586287	A	T	nonsynonymous	86	4	0.044					O		3	0
	ZNF99	19	22940160	C	G	nonsynonymous	64	7	0.099					O		0	0
	FFAR3	19	35850829	G	A	nonsynonymous	76	9	0.106					O	O	2	0
	GPR32	19	51274851	A	C	nonsynonymous	90	5	0.053					O		0	0
	VSTM2L	20	36560072	C	T	nonsynonymous	85	10	0.105					O	O	30	0
	VCX3B	X	8434097	C	A	nonsynonymous	40	4	0.091							0	0
	FTH1P18	X	37061338	C	T	nonsynonymous	81	12	0.129							0	0

Appendix Table S10. CNAs found in patients with medulloblastoma.

Sample	Variant information (WGS)							Driver annotation			RNA-seq
	Chr	Start	End	HSCN1	HSCN2	CNA type	gene annotation	COSMIC cancer gene census	Cancer driver	MB driver	mean FPKM fold change
PD2104	1	10207	249240605	2	1						
	2	11620	243152628	1	1						
	2	243152629	243189358	1	0	Focal LOH	RPL23AP88				
	3	60173	197946427	1	1						
	4	43726	190906081	1	2						
	4	190906082	190986514	1	1						
	5	13078	180905245	1	1						
	6	155130	32454322	2	1						
	6	32454323	32486462	1	1						
	6	32486463	32489150	4	1	Focal amplification					
	6	32489151	32490665	0	1	Focal LOH					
	6	32490666	32497579	1	1						
	6	32497580	32522102	2	1						
	6	32522103	32523235	0	1	Focal LOH					
	6	32523236	32526810	2	1						
	6	32526811	32540031	1	1						
	6	32540032	32550150	2	1						
	6	32550151	32551841	0	1	Focal LOH					
	6	32551842	32557127	3	1	Focal amplification					
	6	32557128	171010389	2	1						
	7	35176	159128639	2	2						
	8	158857	146301439	0	1						
	9	14605	141122067	1	1						
	10	61817	135524731	1	1						
	11	186193	134946454	1	2						
	12	82623	133841504	2	1						
	13	20500092	115109863	1	1						
	14	20301108	107289525	1	2						
	15	21887970	102431958	1	1						
	16	65422	69705	0	1	Focal LOH					
	16	69706	90229218	1	1						
	17	0	81155188	2	2						
	18	14326	49624	1	1						
	18	49625	78017232	1	2						
	19	70879	59118868	1	2						
	20	60000	62965505	1	2						
	21	9428457	48119868	1	1						
	22	18900017	51244551	1	1						
	X	2699502	154930284	0	1						
	1	10207	249240605	1	1						
	2	11520	243189358	1	1						
	3	60173	197946427	1	1						
	4	36516	190962744	1	1						
	5	13278	158254058	1	1						
	5	158254059	180905245	1	0						
	6	157647	171010389	1	1						

	7	26384	140725137	1	1						
	7	140725138	159128639	1	0						
	8	158857	127585797	1	1						
	8	127585798	128231850	1	1						
	8	128231851	128251803	6	1	Focal amplification					
	8	128251804	128251845	5	1	Focal amplification					
	8	128251846	128579132	6	1	Focal amplification	POU5F1B,CASC8				8.429
	8	128579133	128579136	5	1	Focal amplification					
	8	128579137	128656797	6	1	Focal amplification					
	8	128656798	128730193	5	1	Focal amplification					
	8	128730194	128864348	6	1	Focal amplification	MYC	MYC	MYC	MYC	3.79
	8	128864349	128867899	5	1	Focal amplification					
	8	128867900	128883051	6	1	Focal amplification					
	8	128883052	128929953	5	1	Focal amplification					
	8	128929954	128930060	4	1	Focal amplification					
	8	128930061	128932004	5	1	Focal amplification					
	8	128932005	128932031	4	1	Focal amplification					
	8	128932032	128965032	5	1	Focal amplification					
	8	128965033	128965034	4	1	Focal amplification					
	8	128965035	129012930	5	1	Focal amplification	RNVU1-32<br>MIR1205				
PD2105	8	129012931	129040511	6	1	Focal amplification	RNU4-25P				
	8	129040512	129040522	5	1	Focal amplification					
	8	129040523	129134305	6	1	Focal amplification	MIR1207				
	8	129134306	129134414	5	1	Focal amplification					
	8	129134415	129281861	6	1	Focal amplification	RN7SKP226<br>MIR1208				
	8	129281862	130795541	1	1						
	8	130795542	146301439	1	1						
	9	14605	135465323	1	1						
	9	135465324	135889405	2	1						
	9	135889406	141153412	1	1						
	10	61817	135524731	1	1						
	11	195900	74212450	1	1						
	11	74212451	74375379	1	2						
	11	74375380	134946454	1	1						
	12	60729	133841504	1	1						
	13	20500092	115109863	1	1						
	14	20243391	107060338	1	1						
	14	107060339	107289525	2	1						
	15	21887970	102431958	1	1						
	16	60000	90294728	1	1						
	17	0	18970404	0	1						
	17	18970405	81189759	2	1						
	18	21784	78017232	1	1						
	19	212780	59117658	1	1						
	20	60000	62965505	1	1						
	21	9428457	48119868	1	1						
	22	18900017	51234485	1	1						
	X	2835694	154803693	1	0						
	1	10207	249240605	1	1						
	2	11520	243189358	1	1						
	3	60173	50480340	1	1						

	3	50480341	50696608	1	2						
	3	50696609	197946427	1	1						
	4	16200	190958693	1	1						
	5	13544	696892	1	1						
	5	696893	701050	3	1	Focal amplification					
	5	701051	788253	1	1						
	5	788254	792311	1	0	Focal LOH					
	5	792312	121670509	1	1						
	5	121670510	121755149	2	1						
	5	121755150	121755771	3	1	Focal amplification					
	5	121755772	121755842	0	1	Focal LOH					
	5	121755843	121774504	3	1	Focal amplification	SNCAIP				2.759
	5	121774505	121857025	2	1						
	5	121857026	121858044	1	1						
	5	121858045	121858640	4	1						
	5	121858641	180739053	1	1						
	6	151852	171010389	1	1						
	7	17846	95044285	1	1						
	7	95044286	107410549	1	2						
	7	107410749	159128639	2	2						
	8	100703	146301439	0	1						
PD2107	9	14605	141115917	1	1						
	10	61817	135437013	1	1						
	11	194521	134946454	1	1						
	12	77632	4351493	1	1						
	12	4351494	4513773	1	2						
	12	4513774	133841504	1	1						
	13	20500092	115109863	1	1						
	14	20243391	107289525	1	1						
	15	21887970	102461145	1	1						
	16	65422	90229218	1	1						
	17	0	19129655	1	0						
	17	19129656	46005378	1	2						
	17	46005379	46144955	2	2	Focal amplification	COPZ2,NFE2L1 PNPO,CDK5RAP3, PRR15L,MIR152,NFE2L1-DT				0.915
	17	46144956	81189759	1	2						
	18	10063	54010505	1	1						
	18	54010506	78017232	0	1						
	19	212780	59118868	1	1						
	20	60000	35080512	1	1						
	20	35080513	35190516	2	1						
	20	35190517	62965505	1	1						
	21	9422269	48119868	1	1						
	22	18900017	51244551	1	1						
	X	2835694	154871707	1	0						
	1	66226	17054648	1	1						
	1	17054649	17189544	1	0	Focal LOH	MST1L,RNU1-4 CROCCP4,PDE4DIPP9,MIR3675				2.174
	1	17189545	92901931	1	1						
	1	92901932	92960263	1	2						
	1	92960264	249240605	1	1						

	2	11320	121100119	1	1						
	2	121100120	121100344	2	1						
	2	121100345	121102543	0	1	Focal LOH					
	2	121102544	121102932	1	1						
	2	121102933	121102939	2	1						
	2	121102940	121103431	3	1	Focal amplification					
	2	121103432	243189358	1	1						
	3	60173	197896834	1	1						
	4	16200	190941812	1	1						
	5	13544	87599725	1	1						
	5	87599726	87791087	2	1						
	5	87791088	180905245	1	1						
PD2109	6	151531	171010389	1	1						
	7	35176	159128639	1	1						
	8	158857	146301439	1	1						
	9	10000	141115917	1	1						
	10	61817	135524731	1	1						
	11	191612	134946454	1	1						
	12	60729	133833492	1	1						
	13	20500092	115109863	1	1						
	14	20111117	57069670	1	1						
	14	57069671	57401469	2	1						
	14	57401470	107289525	1	1						
	15	21887970	102431958	1	1						
	16	60000	90229218	1	1						
	17	0	81155188	1	1						
	18	10063	78017232	1	1						
	19	212780	59117658	1	1						
	20	60000	62965505	1	1						
	21	9421425	48119868	1	1						
	22	18900017	51228918	1	1						
	X	2835694	154871707	0	1						
	1	10207	37423057	1	1						
	1	37423058	37423209	1	0	Focal LOH					
	1	37423210	37425491	1	1						
	1	37425492	37425631	1	2						
	1	37425632	191804832	1	1						
	1	191804833	191893640	1	2						
	1	191893641	249240605	1	1						
	2	11320	243189358	1	1						
	3	60173	197946427	1	1						
	4	36346	103997720	1	1						
	4	103997721	190986514	1	1						
	5	13544	180905245	1	1						
	6	151531	29290776	1	2						
	6	29290777	170356220	1	1						
	6	170356221	170959010	1	0	Focal LOH	PSMB1,PDCD2 FAM120B,TBP,DLL1,OR4F7P,RPL12P23,LINC01624,MIR4644				0.258
	7	10126	159128639	1	1						

	8	158857	146300929	1	1						
	9	10000	135515011	1	1						
PD2110	9	135515012	135853083	0	1	Focal LOH	GTF3C4,AK8 SPACA9,TSC1 RNU7-21P,MIR548AW	TSC1	TSC1		0.55
	9	135853084	141115917	1	1						
	10	61817	135477858	1	1						
	11	195900	134946454	1	1						
	12	94603	120705647	1	1						
	12	120705648	120819844	1	2						
	12	120819845	133841504	1	1						
	13	20500092	115109863	1	1						
	14	20301108	107289525	1	1						
	15	21943120	75899997	1	1						
	15	75899998	76189748	1	2						
	15	76189749	102521365	1	1						
	16	60000	90294728	1	1						
	17	0	41401179	1	1						
	17	41401180	81182970	1	1						
	18	48777	78017232	1	1						
	19	89293	59118868	1	1						
	20	60000	62965505	1	1						
	21	9428457	48119868	1	1						
	22	18900017	44510365	1	1						
	22	44510366	44511437	0	1	Focal LOH					
	22	44511438	44511822	1	1						
	22	44511823	44511863	2	1						
	22	44511864	51244551	1	1						
	X	2699502	154930284	1	1						

Appendix Table S11. SEdb Samples used for SE and TE annotation

Cancer type	Sample ID	Database	Source	Tissue	Detail
Breast	Sample_01_045	ENCODE	Primary cell	Mammary Gland	mammary-epithelial-cell
Breast	Sample_01_046	ENCODE	Cell line	Mammary Gland	MCF7
Breast	Sample_01_072	ENCODE	Tissue	Breast epithelium	breast-epithelium
Breast	Sample_02_056	NCBI GEO/SRA	Cell line	Mammary Gland	HMEC
Breast	Sample_02_057	NCBI GEO/SRA	Cell line	Mammary Gland	vHMEC
Breast	Sample_02_079	NCBI GEO/SRA	Cell line	Mammary Gland	T-47D_1
Breast	Sample_02_115	NCBI GEO/SRA	Cell line	Mammary Gland	MCF7
Breast	Sample_02_394	NCBI GEO/SRA	Cell line	Mammary Gland	T-47D_2
Breast	Sample_02_399	NCBI GEO/SRA	Cell line	Mammary Gland	SUM159PT-BT-474
Breast	Sample_02_401	NCBI GEO/SRA	Cell line	Mammary Gland	SUM159PT-T47D
Breast	Sample_02_403	NCBI GEO/SRA	Cell line	Mammary Gland	T-47D_untreat
Brain	Sample_02_069	NCBI GEO/SRA	Cell line	Brain	SF268
Brain	Sample_02_138	NCBI GEO/SRA	Tissue	Brain	RT172
Brain	Sample_02_152	NCBI GEO/SRA	Cell line	Brain	U87_EGFRvIII_shFOXG1_FBS
Brain	Sample_02_154	NCBI GEO/SRA	Cell line	Brain	U87_EGFRvIII_shSCR_FBS
Brain	Sample_02_155	NCBI GEO/SRA	Cell line	Brain	U87_EGFRvIII_shSOX9_FBS
Brain	Sample_02_157	NCBI GEO/SRA	Cell line	Brain	U87_EGFRvIII_FBS
Brain	Sample_02_158	NCBI GEO/SRA	Cell line	Brain	U87_EGFRvIII_erlotinib
Brain	Sample_02_160	NCBI GEO/SRA	Cell line	Brain	U87_FBS
Brain	Sample_02_161	NCBI GEO/SRA	Cell line	Brain	U87
Brain	Sample_02_220	NCBI GEO/SRA	Cell line	Brain	BE2C_1
Brain	Sample_02_250	NCBI GEO/SRA	Tissue	Brain	tumormodel3691
Brain	Sample_02_253	NCBI GEO/SRA	Cell line	Brain	BE2C_DMSO
Brain	Sample_02_340	NCBI GEO/SRA	Cell line	Brain	BE2C_2
Brain	Sample_02_407	NCBI GEO/SRA	Cell line	Brain	BT16
Brain	Sample_02_410	NCBI GEO/SRA	Cell line	Brain	BT16_NoDox
Brain	Sample_02_413	NCBI GEO/SRA	Tissue	Brain	GBM_2493
Brain	Sample_02_421	NCBI GEO/SRA	Cell line	Brain	BE2C_3
Brain	Sample_01_019	ENCODE	Primary cell	Brain and spinal cord	astrocyte
Brain	Sample_01_047	ENCODE	In vitro differentiated cells	Embryo	mid-neurogenesis radial-glial-cells
Brain	Sample_01_050	ENCODE	In vitro differentiated cells	Embryo	neural-cell
Brain	Sample_01_051	ENCODE	In vitro differentiated cells	Embryo	neural-progenitor-cell
Brain	Sample_01_064	ENCODE	Cell line	Brain: metastatic site: supra-orbital area	SK-N-MC
Brain	Sample_02_019	NCBI GEO/SRA	Other	Embryo	Neural crest cells
